# Numerical simulation study on pressure response and fluid loss mechanism of coal fracture network under particle temporary plugging

**DOI:** 10.1371/journal.pone.0352681

**Published:** 2026-07-24

**Authors:** Qiushuang Sun, Jian Chen, Liwen Guo, Jiaxuan Han, Yizheng Wang, Xinda Yang, Yanlei Guo, Xiangming Hu, Yee-Chung Jin, Guanhua Ni

**Affiliations:** 1 School of Emergency Management and Safety Engineering, North China University of Science and Technology, Tangshan, China; 2 Tangshan Key Laboratory of Mine Safety and Emergency Management, Tangshan, China; 3 College of Safety and Environmental Engineering, Shandong University of Science and Technology, Qingdao, China; 4 Fac Engn & Appl Sci, University of Regina, Regina, Canada; Northeastern University, CHINA

## Abstract

To address the issue of fluid loss leading to pressure failure when applying temporary plugging fracturing technology from the oil industry to enhance gas recovery in coal mines, and considering the current low efficiency of CO_2_ solidification and utilization, a new method is proposed to inject SC-CO_2_ and hot alkali solution together into coal to produce solidified particles as temporary plugging agents for solidification and utilization of CO_2_ and fracturing transformation of coal. At present, the mechanisms governing pressure increase during temporary plugging with solidified CO_2_ particles, as well as the relationship between internal pore and fracture pressures after plugging and fluid loss rate, remain unclear. Based on CT images, a complex geometric model of interconnected pores and fractures of coal is constructed. The morphology of solidified CO_2_ particles is obtained via microscopic scanning, and conducts numerical simulation research on fluid solid coupling. Clearly define the pressure and flow distribution inside the coal under different temporary plugging positions and injection conditions. Revealing the pressure and fluid loss law inside the coal after temporary plugging. The results show that when particles are temporary plugging at different positions, the average internal pressure in the coal ranges from 8.05 MPa to 12.03 MPa. The fluid loss rate varies from 0.037 cm^3^/s to 0.152 cm^3^/s. Establish equations for the relationship between injection pressure, fracture size, temporary plugging location, internal pressure, and fluid loss rate, with a fitting formula R^2^ of 0.988. Furthermore, an experimental setup and methodology involving supercritical CO_2_ and hot alkali liquor injection were designed to validate the accuracy of the numerical simulation results regarding pressure enhancement and fluid loss under solidified particle plugging. This study provides theoretical support for enhanced coalbed methane recovery and the development of integrated technologies for underground CO_2_ storage and hydraulic control of gas emissions.

## 1. Introduction

Coalbed methane is a coal related energy source with abundant resources. Coal reservoirs generally have characteristics such as low permeability, high adsorption, and strong heterogeneity. Traditional hydraulic fracturing technology faces many challenges in coal reservoir transformation and gas extraction. Temporary plugging involves adding temporary plugging agents to the formation during the construction process, which significantly improves the fracturing effect [1–3]. The solidification and storage of CO_2_ is proposed due to the increase in CO_2_ emissions, which causes environmental protection issues. The main method of CO_2_ solidification and storage is that CO_2_ reacts with minerals or metal oxides to form carbonates [4–6]. Marchetti [7] first proposed CO_2_ geological storage in 1977. The CO_2_ injected into the underground reservoir undergoes slow chemical reactions with water and mineral components in the underground reservoir to generate carbonate minerals such as MgCO_3_ and CaCO_3_ to achieve the permanent stable storage of CO_2_ [8–10]. In 1990, Seifritz [11] first proposed the concept of CO_2_ mineralization. Subsequently, scholars based on this concept used the carbonization reaction between minerals and CO_2_ and metal oxides to generate stable carbonates to achieve the mineral storage of CO_2_ [12–19]. With the continuous deepening of research on CO_2_ storage, the utilization of CO_2_ has received great attention [20–22]. The resource utilization and storage of CO_2_ has become an important path to achieve the “dual carbon” strategic goals. Supercritical CO_2_ (SC-CO_2_) has low viscosity, strong permeability, and good injection effect in coal seams, and shows unique advantages in fields such as coal seam gas displacement and coal reservoir fracturing modification. However, the existing SC-CO_2_ fracturing technology mainly focuses on its physical and mechanical effects as a fracturing medium, and pays insufficient attention to the solidification and utilization of CO_2_ after injection into coal rock. The efficiency of CO_2_ solidification and storage is low, and the potential of CO_2_ resource utilization in the fracturing process has not been fully exploited.

Our team innovatively proposed using the SC-CO_2_ injected into the coal and the solidification particles produced by the hot alkali liquor as the temporary plug for the fracturing process, in order to develop a new method for solidifying and utilizing CO_2_ and modifying the coal through fracturing. At the same time, CaCO_3_ was injected into the internal connected pores and fractures of the coal as the temporary plug to achieve temporary plugging and pressure retention. If SC-CO_2_ and hot alkali liquor were injected separately into the connected pores and fractures of the coal, it would simultaneously realize the solidification of CO_2_ and the utilization of the fracturing process.

Early studies on particle temporary plugging mainly focused on selecting particle diameters to achieve the optimal temporary plugging effect. Various theories such as the “ideal filling theory (d_1/2_ theory)” [23], the “one-third” bridge theory [24], single-particle and multi-particle bridge models [25] were proposed. Additionally, many scholars based on the ideal filling theory conducted research on the selection of temporary plugging particles, proposing rules such as d_50_, d_90_ rules, and Vickers rules [26–28]. Since the 20th century, the mechanism of temporary plugging pressure increase in fracturing cracks has become a key research topic for scholars worldwide.The three most classic pressure increase models are the “stress cage” [29] model, the “fracture closure stress” [30] model, and the “fracture expansion resistance” [31] model, which qualitatively evaluate the fracture initiation mechanism of temporary plugging fracturing from the aspects of fracture mouth temporary plugging, fracture interior temporary plugging, and fracture tip temporary plugging.

During the temporary plugging process, pressure distribution and pressure variations play a decisive role in determining the effectiveness of temporary plugging. Meanwhile, the temporary plugging effect of temporary plugging particles influences fluid flow within fractures and fluid loss in pores. Currently, the rules of pressure change and fluid loss in the fractures after the solidification of CO_2_ particles for temporary plugging coal are not clear. Therefore, it is necessary to conduct in – depth research on this.

The temporary plugging caused by temporary plugging particles is a highly complex physical process [32–34]. The pressure distribution and fluid flow distribution inside the coal fractures after temporary plugging are difficult to be directly and accurately reflected through experiments. The pressure field and flow field cannot be continuously statistically analyzed, and the data at each spatial point cannot be continuously statistically analyzed. Therefore, numerical simulation methods are selected to study this.

In the research methods of temporary plugging pressure simulation, the Euler-Lagrange method overcomes the computational limitation of the Euler-Euler method, which treats particles as a pseudo-fluid phase. It has become the main method for scholars to study the migration of temporary plugging particles [35–40].

However, in the related numerical simulation studies on geometric modeling, the pores of the rock mass are usually ignored, which cannot reflect the actual geometric shape of the coal fractures [41–43].

Therefore, this paper uses CT technology to establish a complex connected pore-fracture geometric model of coal, investigates the pressure and flow distribution in coal fractures after temporary plugging by solidified CO_2_ particles, and reveals the pressure variation patterns and fluid loss patterns in micro- and macro-connected pore-fractures of coal after temporary plugging by plugging particles.

In addition, an SC-CO_2_ - hot alkali injection test system and an experimental method were designed to simulate the generation of SC-CO_2_ solidified particles in the coal sample. The SC-CO_2_-hot alkali injection test mainly uses the SC-CO_2_-hot alkali injection test system to inject SC-CO_2_ and hot alkali solution into the coal sample. It conducts coal injection experiments of SC-CO_2_-hot alkali under different injection conditions to verify the accuracy of the pressure increase rules and fluid loss rules in the numerical simulation results under the temporary plugging effect of SC-CO_2_ solidified particles.

This study can provide scientific basis and data support for the temporary plugging and pressure control issues faced by coal mines in applying temporary plugging and fracturing technology to increase coalbed methane production.

## 2. Construction of microscopic interconnected pore-fracture geometric model of coal

Uniform coal samples were prepared as the scanned samples. The German Phoenix V|tome|x m CT system was used to scan the coal samples. The experimental scanning parameters are shown in Table 1. FOD is the distance between the radiation source and the sample, in millimeters; FDD is the distance between the radiation source and the detector, in millimeters; the ratio of FDD to FOD (FDD/FOD) is the geometric magnification.

**Table 1:** Basic experimental parameters of the CT scan of the coal sample.

Parameter	Power(W)	Voltage (kV)	Electric current (μA)	Exposure time (ms)	Resolution(μm)	FOD(mm)	FDD(mm)	Geometric magnification
Value	12	50	80	500	1.5	49.54	810.55	16.36

**Fig 1:**
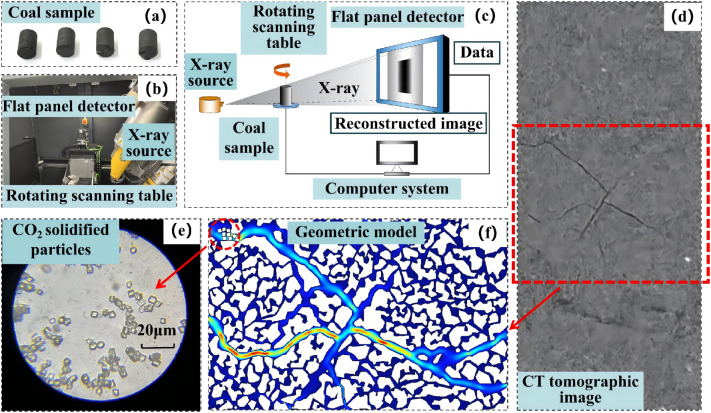
Geometric model construction process.

The process of modeling the micro-macroscopic connected pore and fracture geometry of coal is shown in Fig 1. Fig 1(a) shows the selection of coal samples for CT scanning. The coal samples are placed on the rotating scanning platform shown in Fig 1(b). The principle of CT scanning is shown in Fig 1(c). The CT scanning experiment ultimately yielded 1600 two-dimensional CT cross-sectional images.

The CT scanning experiment obtained a two-dimensional CT grayscale image of the coal sample, which was imported into Avizo for processing. Image processing techniques such as filtering, noise reduction, and threshold segmentation were applied to obtain a binary image.

The ultimate goal of noise reduction is to provide high signal-to-noise ratio data for coal and rock fracture network modeling, while avoiding the loss of microstructural information due to excessive smoothing. Selecting a median filter to achieve image denoising not only removes noise from the image but also preserves the edge features of the image, resulting in good denoising and image restoration effects. Based on the bimodal distribution characteristics of the two-phase grayscale histogram of coal rock matrix and pores, a globally optimal bimodal adaptive threshold iteration algorithm is employed, combined with cross-checking of multiple slices within the same batch, to determine the segmentation threshold. Avoid the upper and lower end faces of the coal sample, the edge processing disturbance zone, and the local abnormal high-density mineral inclusion zone. Select typical cross sections from the upper, middle, and lower layers at equal intervals along the axial direction of the coal sample. Images with obvious fracture structures were selected for processing, and the final geometric structure of the micro-macroscopic connected pores and fractures of coal was obtained, as shown in Fig 1(f).

To obtain the real geometric shape of CO_2_ solidification particles, an experiment on the carbonization reaction between CO_2_ and a saturated Ca(OH)_2_ solution to form precipitates was conducted. The CaCO_3_ obtained from the carbonization reaction is called lightweight CaCO_3_ or precipitated CaCO_3_. The chemical reaction equation is as shown in Equation (1):


$$\begin{array}{c} Ca(OH{)}_{\text{2}}+CO_{\text{2}}=CaCO_{\text{3}}↓+H_{\text{2}}O \end{array}$$ (1)

After the reaction was completed, samples were taken for observation and analysis. The morphology of the precipitated particles is shown in Fig 1(e). The precipitated particles generated during the reaction were mainly square-shaped. Therefore, a square-shaped geometric model of temporary plugging particles was established in the numerical simulation.

## 3. Governing equations

### 3.1. Basic assumptions of the calculation model

Particle plugging hydraulic fracturing is a complex multi-field coupled process. The following basic assumptions are adopted in the numerical simulation: temporary plugging particles are assumed to be homogeneous, square elastic particles.

(1) The fluid is assumed to be Newtonian and incompressible.
(2) Temporary plugging fracturing involves multiple processes such as particle migration, pressure maintenance, and fracture initiation. Particle migration has been investigated in the paper “Migration of CO_2_‑solidified temporary plugging particles used for temporary plugging fracturing in microscopic interconnected pores and fractures of coal”. This study focuses on plugging mechanisms, and fracture initiation is not considered.
(3) The pre-stress, heat exchange and coal swelling are neglected, and coal is treated as a linear structural material.

### 3.2. Governing Equations

Under the assumption that coal has homogeneity, linear elasticity, and other characteristics, the deformation control equation of coal is expressed by the equilibrium differential equation, physical equation, and geometric equation, in which the equilibrium differential equation is expressed as


$$\begin{array}{c} \rho_{\text{c}}\frac{\partial^{\text{2}}{\vec{u}}_{\text{c}}}{\partial t}+d_{c}\frac{\partial {\vec{u}}_{\text{c}}}{\partial t}-\nabla \cdot \tilde{\sigma }=\vec{f}, \end{array}$$ (2)

where $\rho_{\text{c}}$ is density of coal, kg/m^3^; ${\vec{u}}_{c}$ is coal displacement, m; $d_{c}$ is the damping coefficient, m/s; $\tilde{\sigma }$ is the surface stress of coal, N; and $\vec{f}$ is the volume force, N.

The physical equations of coal deformation satisfy the generalized Hooke’s law. This law mainly describes the constitutive relationship between stress and strain, which can be expressed as


$$\begin{array}{c} S_{c}-\left(S_{\text{0}}+S_{ext}+S_{q}\right)=C:\left[\varepsilon_{c}-\left(\varepsilon_{\text{0}}+\varepsilon_{th}+\varepsilon_{hs}+\varepsilon_{pl}+\varepsilon_{cr}\right)\right], \end{array}$$ (3)

where $S_{c}$ is the stress, N; $S_{\text{0}}$ is the prestress, N; $S_{ext}$ is the external stress, N; $S_{q}$ is the viscous stress, N; C is the elastic matrix; $\varepsilon_{c}$ is the elastic strain of coal; $\varepsilon_{\text{0}}$ is the prestrain; $\varepsilon_{th}$ is the thermal strain; $\varepsilon_{hs}$ is infiltration expansion; $\varepsilon_{pl}$ is plastic strain; $\varepsilon_{er}$ is creep.

The resultant force of temporary plugging particles is summarized as the sum of gravity, elastic force, damping force, friction force, adhesion force, and fluid drag force, expressed by Formula (4):


$$\begin{array}{c} F_{a}=F_{g}+F_{ela}+F_{dam}+F_{fric}+F_{coh}+F_{hyd}, \end{array}$$ (4)

where $F_{a}$ is the combined external force of temporary plugging particles; $F_{g}$ is the gravity of the particles; $F_{ela}$ is the elastic force; $F_{dam}$ is the damping force; $F_{fric}$ is the friction force; $F_{coh}$ is adhesion; $F_{hyd}$ is the drag force of fluid.

The motion equation of temporary plugging particles satisfies Newton’s second law, and the motion of particles in the interconnected pores and fractures of coal is expressed in two ways: translation and rotation. With a single particle taken as the research object, its motion equation can be expressed as follows:


$$\begin{array}{c} m_{p}\frac{\partial u_{p}}{\partial_{t}}=F_{g}+F_{p}+F_{f}+F_{c}, \end{array}$$ (5)

$$\begin{array}{c} {\text{I}}_{p}\frac{\partial \omega_{p}}{\partial_{t}}={\text{M}}_{p}, \end{array}$$ (6)

where $m_{p}$ is the mass of particles, kg; $F_{p}$ is the particle force on the particle, N; $F_{f}$ is the fluid force on the particle, N; $F_{c}$ is the coal force on the particles, N; ${\text{I}}_{p}$ is the moment of inertia of the particle, kg/m^2^; $\omega_{p}$ is the rotational angular velocity of the particle, rad/s; ${\text{M}}_{p}$ is the particle bending moment, generated by tangential friction and tangential collision force, N·m.

## 4. Numerical simulation scheme and parameter setting

**Fig 2:**
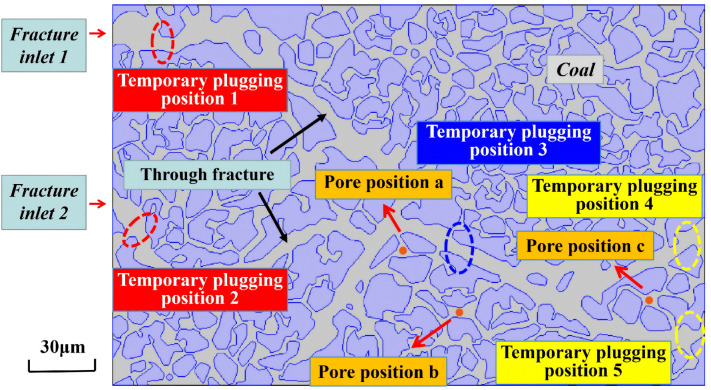
Selection of plugging locations.

Fig 2 shows the micro-macroscopic geometric model of the connected pores and fractures in coal.

In the studied geometric model, five temporary plugging positions were respectively selected at the entrance of the fracture, the middle position inside the fracture, and the end of the fracture as the research objects, namely temporary plugging positions 1–5. Near temporary plugging position 3, three pore positions, a, b, and c, were selected. Among them, pore position a is located before temporary plugging position 3, while pore positions b and c are located after temporary plugging position 3.

The parameters of the temporary plugging positions 1–5 are shown in Table 2.

**Table 2:** Plugging position parameters.

Plugging position	Position 1	Position 2	Position 1, 2	Position 3	Position 4	Position 5	Position 4, 5
Equivalent diameter(μm)	9.32	7.88	17.20	7.80	6.54	6.69	13.23
Length from the inlet(μm)	33.56	17.97	25.77	221.95	378.48	391.29	384.89

Specifically: temporary plugging positions 1 and 2 indicate simultaneous temporary plugging at position 1 and position 2; temporary plugging positions 4 and 5 indicate simultaneous temporary plugging at position 4 and position 5. The equivalent diameters of temporary plugging positions 1 and 2 are the sum of the diameters of position 1 and position 2; The equivalent diameters of temporary plugging positions 4 and 5 are the sum of the diameters of position 4 and position 5. The distances from temporary plugging positions 1 and 2 to the inlet are the average of the distances from position 1 and position 2 to the inlet; The distances from temporary plugging positions 4 and 5 to the inlet are the average of the distances from position 4 and position 5 to the inlet.

The numerical simulation scheme is specifically shown in Table 3.

**Table 3:** Numerical simulation scheme.

Research content	Pressure and flow distribution	Pressure increase rule	The law of fluid loss	
			Fluid loss law after temporary plugging	Fluid flow patterns at pore positions
Injection pressure(MPa)	8MPa	6–14MPa		8MPa
Plugging position	Position 1–5; Position 1, 2; Position 4, 5			Position a, b, c
Injection particles	Temporary plugging particle group			

The settings of relevant parameters for the numerical simulation are shown in Table 4.

**Table 4:** Parameters of numerical simulation.

Parameter	Value
Hydrodynamic viscosity (Pa·s)	1.01e-3
Fluid density (kg/m^3^)	1
Particle density (kg/m^3^)	2700
Young’s modulus of particle (GPa)	2.5
Poisson ratio of particle (1)	0.3
Young’s modulus of coal (GPa)	0.1
Poisson ratio of coal (1)	0.314
Coal density (kg/m^3^)	1400

The fluid uses pure water as the medium, referring to the physical property standards of water at room temperature and pressure, and combining with the subtle environmental differences of simulated working conditions, the fluid dynamic viscosity is determined to be 1.01e-3 Pa·s, and the fluid density is 1 kg/m^3^. The density of CO_2_ solidified particles is 2700 kg/m^3^. Based on the in-situ coal bulk density test results, the overall density of coal with pore structure is determined to be 1400 kg/m^3^. Based on the results of uniaxial and triaxial mechanical tests on coal, as well as simulation experience of similar coal seams, combined with the deformation characteristics of primary coal in the study area, the Young’s modulus of coal is determined to be 3.5 GPa, and the Poisson ratio of coal is taken as 0.314, which can accurately characterize the deformation law of coal and rock under stress and adapt to the solid fluid coupling simulation conditions of this study.

### 4.1. SC-CO_2_-hot alkali injection verification experiment

Experimental equipment: This experiment utilizes a self-developed SC-CO_2_-hot alkali injection test system. Different from conventional hydraulic fracturing experiments, during the experiment, SC-CO_2_ and hot alkali solution need to be injected into the coal sample successively. The SC-CO_2_- hot alkali injection test system mainly comprises an SC-CO_2_ injection system, an alkali injection system, a coal sample holder, a triaxial pressure control system, and a temperature control system.

The coal sample used in the test is taken from Xinjiang, and the coal type is bituminous coal. Bituminous coal is a coal with a medium degree of metamorphism, lying between lignite and anthracite.

Its appearance is gray – black to black, with luster, and the fracture is usually conchoidal or uneven. The chemical composition of bituminous coal is between that of peat and anthracite, with a carbon content ranging from 75% to 90%, and it also contains certain amounts of hydrogen, oxygen, nitrogen, and sulfur.

Experimental method: According to the requirements of the experiment, 10 cylindrical coal samples with a diameter of 25 mm and a length of 80 mm were prepared. During the SC-CO_2_-hot alkali injection test, considering the critical pressure of SC-CO_2_, the injection pressure was set between 7.5 MPa and 11.5 MPa. When injecting the alkali solution, to prevent the backflow of SC – CO_2_, the injection pressure of the alkali solution was set to be higher than that of SC – CO_2_, ranging from 8 MPa to 12 MPa. The axial pressure and confining pressure were higher than the injection pressure, both set at 14 MPa. The test temperature was higher than the critical temperature of SC- CO_2_, and considering the heat loss during the experiment, it was set at 45°C. Remove excess gas or liquid from the pipeline before injection. First, pressurize the CO_2_, open the injection valve of the coal sample holder, and inject SC-CO_2_ into the interior of the coal sample. When the pressure at the injection port remains stable, close the SC-CO_2_ injection valve. Open the liquid outlet valve in front of the coal sample holder inlet and discharge the remaining CO_2_ in the pipeline. Inject alkaline solution into the coal sample according to the experimental pressure setting. After the solidified particles form a stable accumulation, the pressure inside the coal sample and injection port remains stable, and the injection of alkali solution is stopped. Inject and record the inlet pressure of the gripper and the readings of the flow meters at the inlet and outlet of the coal sample gripper during the SC-CO_2_ and alkali solution processes. At the end of the experiment, unload the pressure and take out the coal sample.

The test scheme is shown in Table 5 as follows:

**Table 5:** SC-CO_2_-hot alkali injection scheme.

Parameter	Temperature(°C)	Axial compression (MPa)	Confining pressure (MPa)	SC-CO_2_ injection pressure (MPa)	Alkali injection pressure (MPa)
Value	45	14	14	7.5、8.5、9.5、10.5、11.5	8、9、10、11、12

## 5. Result and discussion

### 5.1. Pressure distribution and pressure variation patterns of the solidified particles in temporary plugging the connected pores and fractures

The pressure distribution within the pore fissures when particles block different positions can be obtained from the pressure contour map. Moreover, numerical simulation can provide the variation of the average pressure within the pore fissures over time during the particle temporary-blocking process. The change in the average pressure within the pore fissures reflects the influence of particle temporary plugging on the internal pressure of the coal.

(1) Pressure distribution within the coal pores and fractures after the temporary temporary plugging with particles

**Fig 3:**
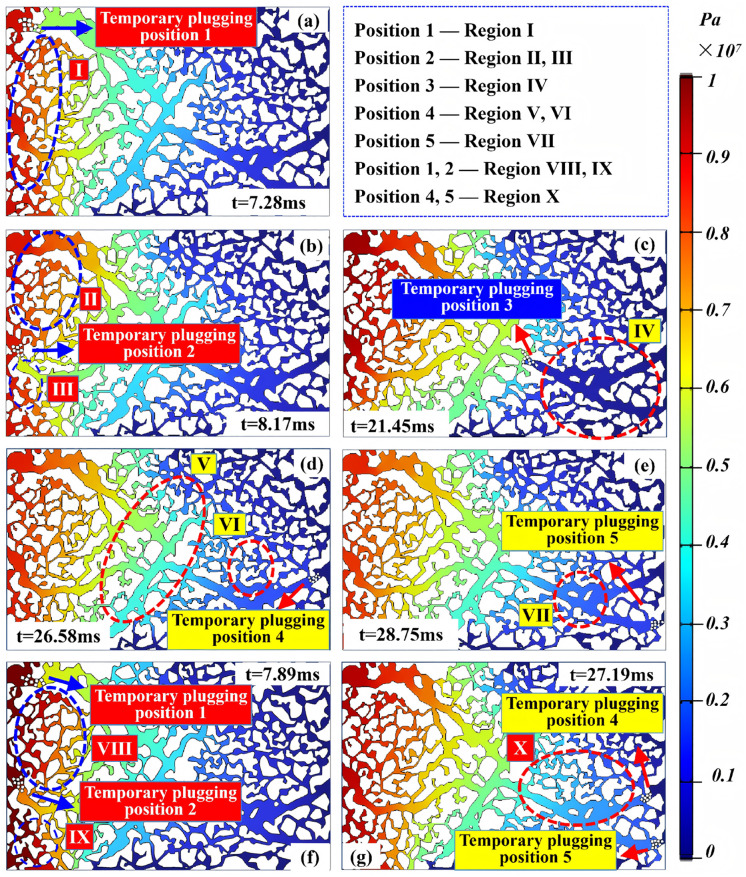
The pressure distribution within the pores and fractures when particles are blocked at different positions.

Set the injection pressure to 8 MPa. When the blocking particles are placed at blocking positions 1–5 respectively, the pressure distribution inside the fracture channels is shown in Fig 3. As can be seen from Fig 3, the pressure distribution inside the fracture channels decreases from the inlet side to the outlet side.

From Fig 3 (a), it can be seen that when the particles are stably blocked at blocking position 1, the pressure of the through-fracture channel where the temporary plugging particles are located decreases, and the pressure inside the fracture channels mainly concentrates on the inlet side of the fracture channels and fracture channel region I.

From Fig 3 (b), it can be seen that when the particles are stably blocked at blocking position 2, the pressure of the through-fracture channel where the temporary plugging particles are located decreases, and the pressure inside the fracture channels mainly concentrates on the inlet side of the fracture channels and fracture channel regions II and III.

**Fig 4:**
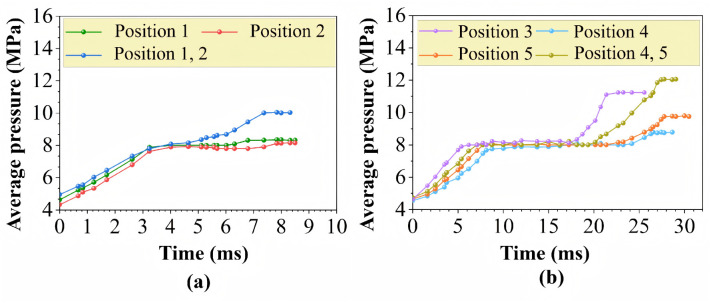
The curve showing the average internal pressure of the fissure at different temporary plugging positions changing over time.

When the particles are blocked at blocking position 3, the pressure distribution inside the fracture channels is shown in Fig 3 (c), and the pressure distribution inside the fracture channels decreases from the side close to the inlet of the fracture channels to the outlet side. The effect of the particle blocking on the pressure at fracture channel region IV is significant, and the pressure is significantly greater than the pressure at the corresponding positions in Figs 4 (a), (b), (d), and (e).

From Fig 3 (d), it can be known that when the particles are stably blocked at the blocking position 4, the pressure at the fracture channel region V in Fig 3 (d) is significantly greater than the corresponding positions in Fig 3 (a) and (b), and the pressure at the fracture channel region VI in Fig 3 (d) is significantly greater than the corresponding position in Fig 3 (e). From Fig 3 (e), it can be known that when the particles are stably blocked at the blocking position 5, the pressure at the fracture channel region VII in Fig 3 (e) is significantly greater than the corresponding position in Fig 3 (d).

When the particles are simultaneously sealed at positions 1 and 2, as well as at positions 4 and 5, the pressure distribution within the pore fissures is shown in Fig 3(f) and (g). From Fig 3(f), it can be observed that when the particles are stably sealed at positions 1 and 2, the pressures in the two through-fissure paths decrease, and the pressure within the pore fissures is mainly concentrated at the entrance side of the fissures and in regions VIII and IX of the pore fissures in Fig 3(f). Moreover, when the particles are simultaneously sealed at positions 1 and 2, the pressure in the pressure-concentration region is significantly higher than when the particles are sealed at position 1 in Fig 3(a) or at position 2 in Fig 3(b).

From Fig 3(g), when the particles are stably sealed at positions 4 and 5, the pressure distribution within the pore fissures decreases from the entrance side to the exit side. The pressure at region X in Fig 3(g) is significantly higher than that when the particles are sealed at position 4 in Fig 3(d) or at position 5 in Fig 3(e). The numerical simulation results indicate that the pressure distribution within the coal varies when the temporary plugging particles are sealed at different positions.

(2) The curve depicting the average pressure within the fracture channels at different temporary plugging positions over time

Set the injection pressure to 8 MPa, organize the data, and draw a comparison chart of the changes in the average pressure inside the pore fractures over time when the temporary plugging particles are blocked at different positions under the same injection pressure. The chart is shown in Fig 4. In Fig 4 (a), the average pressure inside the pore fractures changes over time when the solidified particles are blocked at position 1, position 2, and at positions 1 and 2 simultaneously. In Fig 4 (b), the average pressure inside the pore fractures changes over time when the solidified particles are blocked at position 3, position 4, position 5, and at positions 4 and 5 simultaneously.

From Fig 4, it can be observed that during the process of temporarily blocking particle movement and temporary plugging the pores and fractures in the coal, the average pressure inside the pores and fractures of the coal gradually increased at the initial stage as the inlet fluid was injected. When the injected fluid filled the interior of the coal pores and fractures, the average pressure inside the pores and fractures remained relatively stable. At each temporary plugging position, the stable value of the average pressure was basically the same, approximately equal to the injection pressure of 8 MPa.

When the temporary plugging particles approached the temporary plugging position of the pores and fractures, the average pressure inside the pores and fractures continued to gradually increase until the particles were stably blocked, and then the average pressure tended to stabilize. The average pressure inside the pores and fractures varied when the temporary plugging particles were blocked at different temporary plugging positions.

When the temporary plugging particles were blocked at positions 4 and 5 simultaneously, the stable value of the average pressure inside the pores and fractures was the largest, reaching 12.03 MPa; when blocked at position 3, the average pressure was 11.23 MPa; when blocked at positions 1 and 2 simultaneously, the average pressure was 10.50 MPa; when blocked at position 5, the average pressure was 9.75 MPa; when blocked at position 4, the average pressure was 8.78 MPa; when blocked at position 2, the average pressure was 8.21 MPa; and when blocked at position 1, the average pressure was 8.05 MPa.

The numerical simulation results indicate that the temporary plugging position of the solidified temporary plugging particles affects the average pressure inside the coal pores and fractures after the temporary plugging particles complete the temporary plugging. By comparing the stable values of the average pressure after temporary plugging at different positions, it can be found that for temporary plugging positions close to the inlet, when the temporary plugging particles are blocked at a single position, the stable value of the average pressure inside the pores and fractures is lower than that when the temporary plugging particles are blocked at multiple positions.

For example, when the temporary plugging particles are blocked at positions 1 and 2 simultaneously, the stable value of the average pressure after temporary plugging is less than that when the temporary plugging particles are blocked at positions 4 and 5 simultaneously. When blocked at positions 4 and 5 simultaneously, the stable value of the average pressure after temporary plugging is greater than that when the temporary plugging particles are blocked at positions 1 and 2 simultaneously.

In addition, from the temporary plugging pressure values at positions 5 > 4 > 1 > 2, and from the fact that the temporary plugging pressure values when positions 4 and 5 are simultaneously blocked> when position 3 is blocked> when positions 1 and 2 are blocked simultaneously, it can be seen that the closer the temporary plugging particle temporary plugging position is to the inlet, the smaller the stable value of the average pressure inside the coal pores and fractures after temporary plugging. When the temporary plugging particle temporary plugging position is farther from the inlet, the average pressure increase effect of the temporary fracturing is better.

(3) The variation law of internal pressure within the pores and fractures under different injection pressures

**Fig 5:**
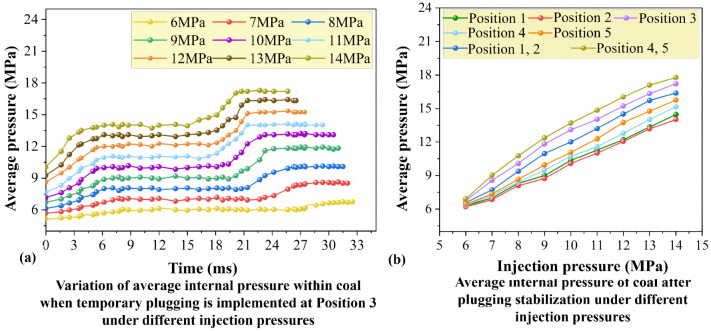
The variation law of internal pressure within the pores and fractures under different injection pressures.

The injection pressure has an impact on the temporary plugging and fracturing pressurization effect. Meanwhile, the experimental pressure is set between 8 MPa and 12 MPa. In the numerical simulation, the injection pressure range is set slightly larger than the experimental pressure setting range, which is from 6 to 14 MPa. Position 3 is selected as the research object, and the curves of the average internal pressure of the fractures at position 3 during the temporary plugging process with different injection pressures over time are obtained, as shown in Fig 5 (a).

Based on the numerical simulation results, it can be concluded that under an injection pressure ranging from 6 to 14 MPa, during the process of the temporary plugging particles’ migration and temporary plugging of the coal pores and fractures, the average pressure inside the coal pores and fractures gradually increases in the initial stage as the inlet fluid is injected. When the injected fluid fills the interior of the coal pores and fractures, the average pressure inside the pores and fractures remains relatively stable. The stable values are all close to the injection pressure. With the change of the injection pressure, the time when the average pressure inside the pores and fractures changes varies. As the injection pressure increases, the time for the temporary plugging particles to complete the temporary plugging gradually decreases. When the temporary plugging particles complete the temporary plugging, the average pressure increases from approximately 2.75 MPa under an injection pressure of 6 MPa to about 13.21 MPa under an injection pressure of 14 MPa. Under the injection pressure range of 6–14 MPa, the curves of the average pressure values inside the pores and fractures after the temporary plugging particles are sealed at different positions and under different injection pressures are plotted, as shown in Fig 5(b). From the figure, it can be seen that when the particles are sealed at a certain position, the larger the injection pressure, the greater the average pressure after temporary plugging. Under the same injection pressure, the average pressure after temporary plugging increases with the increase in the distance between the temporary plugging position and the injection inlet, and the greater the injection pressure, the more obvious the difference in the average pressure of the particles sealed at different temporary plugging positions.

### 5.2. Distribution of fluid within the connected pores after solidification of particles and the law of fluid loss from the outlet

The internal flow field of coal fractures is affected by the blocking particles that temporarily seal the fractures. This is because the pressure difference is the driving force for fluid flow, which does work to overcome resistance. The flow velocity of the fluid inside the coal fractures depends on the pressure difference within the coal. The blocking of the particles at different positions leads to differences in the pressure distribution within the coal, and simultaneously alters the pressure differences in different regions of the coal, resulting in differences in the flow field of the fluid in the coal fractures and pores.

(1) Flow distribution within the pores and fractures of coal and rock after the temporary temporary plugging with particles

**Fig 6:**
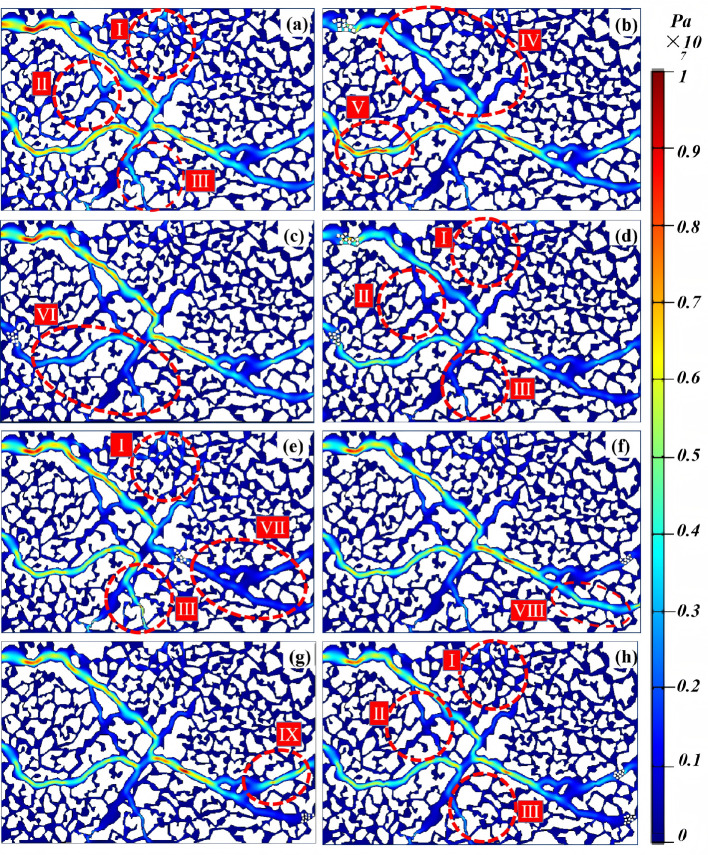
The distribution of the internal flow field within the coal pores and fractures after particle blocking.

The temporary plugging of particles led to changes in the internal flow field within the pores and fractures of the coal, as shown in Fig 6.

Fig 6(a) shows the fluid flow inside the pore fractures when there are no temporary plugging particles. The fluid flows from the inlet to the outlet of the pore fractures, and the fluid velocities at regions I, II, and III of the pore fracture area are relatively high. Fig 6(b) depicts the fluid flow inside the coal pore fractures when the particles are stably blocked at position 1. The fluid velocity within the through fractures blocked by the particles decreases significantly. As shown in Fig 6(b), the fluid velocity at region IV of the pore fracture area is significantly lower than that at the corresponding position in Fig 6(a), and the fluid velocity in region V of the fracture increases.

Fig 6(c) shows the fluid flow inside the coal pore fractures when the particles are blocked at position 2. The fluid velocity within the through fractures blocked by the particles decreases significantly. As shown in Fig 6(c), the fluid velocity at region VI of the pore fracture area is significantly lower than that at the corresponding position in Fig 6(a), and the fluid mainly flows from the inlet 1 to the outlet direction.

Fig 6(d) shows the fluid flow inside the coal pore fractures when the particles are blocked at both positions 1 and 2 simultaneously. As shown in the figure, the fluid velocity inside the coal fractures decreases. In Fig 6(d), the fluid velocities at regions I, II, and III of the pore fracture area are significantly lower than those at the corresponding positions in Fig 6(a).

Fig 6(e) shows the fluid flow inside the coal pores and fractures when the particles are stably blocked at the temporary plugging position 3. Due to the particle blocking, the flow velocity in the fracture region VII is significantly reduced. The flow velocities in the fracture regions I and III are significantly higher than those at the corresponding positions in Figs 6(b), (c), and (d). This is because when the particles are blocked at the temporary plugging position 3, the fluid cannot flow out through the main fracture outlets, resulting in an increase in the flow velocities in the fracture regions I and III near the pore outlets.

Fig 6(f) shows the fluid flow inside the coal pores and fractures when the particles are stably blocked at the temporary plugging position 4. The flow velocity of the fluid in the through – fractures blocked by the particles is significantly reduced, and the flow velocity in the fracture region VIII is significantly higher than that when the particles are blocked at other positions.

Fig 6(g) shows the fluid flow inside the coal pores and fractures when the particles are stably blocked at the temporary plugging position 5. The flow velocity of the fluid in the through – fractures blocked by the particles is significantly reduced, and the flow velocity in the fracture region IX is significantly higher than that when the particles are blocked at other positions.

Fig 6(h) shows the fluid flow inside the coal pores and fractures when the particles are simultaneously blocked at the temporary plugging positions 4 and 5. As shown in the figure, the flow velocities in the fracture regions I, II, and III are significantly lower than those at the corresponding positions in Fig 6(a).

The numerical simulation results indicate that the temporary plugging position of the temporary plugging particles affects the flow distribution of the fluid inside the coal pore and fracture.

(2) Fluid flow pattern in pore positions

**Fig 7:**
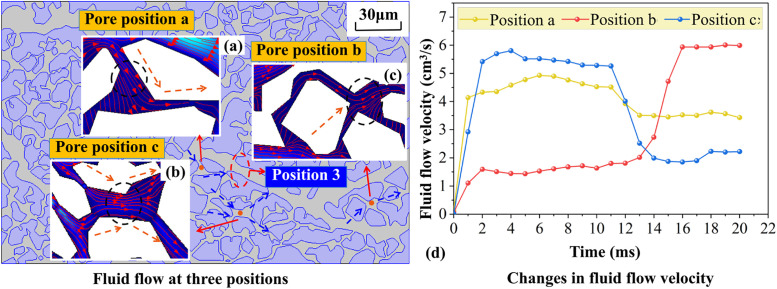
Fluid flow at three pore positions.

The coal seam mainly consists of two types of permeable media: fractures and coal matrix. The fluid flow within the coal fractures is strong, while the fluid flow within the coal matrix is weaker than that in the fractures. However, the volume of the coal matrix is much larger than that of the fractures, and the pores within the matrix have a significant impact on the fluid loss of the fluid. Taking the temporary plugging position 3 as the research object, three pore positions were selected, as shown in Fig 7.

The characteristics of the three pore positions are as follows: Pore position a is located before the temporary plugging position 3. The fluid flow direction at this position is shown in Fig 7 (a), where the fluid flows from the fractures to pore position a and then to the fractures. Both the inflow and outflow positions are located before the temporary plugging position 3. Pore position b is located after the temporary plugging position 3. The fluid flow direction at this position is shown in Fig 7 (b), where the fluid flows from the pores and fractures before the temporary plugging position 3 to the pores and fractures after the temporary plugging position 3. Pore position c is located after the temporary plugging position 3. The fluid flow direction at this position is shown in Fig 7 (c).

The fluid flows from the fracture to pore position c and then to the model outlet. Both the inflow and outflow positions are located after temporary plugging position 3. The variation of fluid flow velocity at pore position a, pore position b, and pore position c with time is shown in Fig 7(d).

From the figure, it can be seen that the fluid flow velocity at pore position a, pore position b, and pore position c increases to a certain value at the initial injection time, and the velocity magnitude remains relatively stable within a certain period. Moreover, the velocity at pore position c is greater than that at pore position a, which is in turn greater than that at pore position b. As the temporary plugging particles move towards the throat area, the fluid flow velocity at pore position a and pore position c gradually decreases, while the fluid flow velocity at pore position b gradually increases, until the particle cluster forms a stable seal.

After the temporary plugging is completed, the fluid flow velocity at pore position b is greater than that at pore position a, which is in turn greater than that at pore position c. The reason for this is that the pressure difference serves as the driving force for fluid flow. The fluid flow velocity within the pores and fractures of the coal depends on the pressure difference inside the coal. The flow field of the fluid in the pores varies according to different pore positions.

The inflow and outflow positions at pore position a and pore position c are located before temporary plugging position 3. The influence of the temporary plugging particles on the pressure difference at the fluid inlet and outlet is relatively small. Thus, the fluid flow velocity at pore position a changes little after the temporary plugging particles form a seal.

The inflow and outflow positions at pore position c are located after temporary plugging position 3, and the fluid mainly flows into the fractures at this position. After the temporary plugging particles form a seal, the fluid flow velocity in the fractures after temporary plugging position 3 decreases significantly, and the pressure difference at the fluid inlet and outlet of pore position c is small. Therefore, the fluid flow velocity at pore position c decreases significantly after the temporary plugging particles form a seal.

The fluid at pore position b flows from the fracture in the pore position before temporary plugging position 3 to the fracture after temporary plugging position 3. When the temporary plugging particles form a seal, the pressure difference before and after temporary plugging position 3 is large. So, the fluid flow velocity at pore position b increases significantly after the temporary plugging particles form a seal.

(3) The variation law of fluid loss rate over time during temporary plugging at different positions

**Fig 8:**
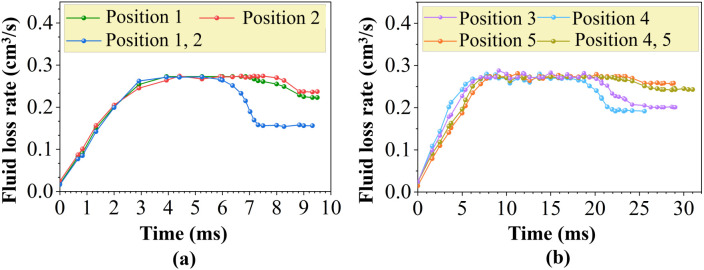
The curves depicting the variation of fluid loss rate at different positions over time.

Set the injection pressure to 8 MPa. During the process of temporary plugging the fractures in the coal with the curing plug particles at different positions, the curve of the coal fluid loss rate varying with time is shown in Fig 8.

Specifically, Fig 8(a) shows the curve of the coal fluid loss rate varying with time when the curing particles are respectively placed at position 1, position 2, or at positions 1 and 2 simultaneously. Fig 8(b) shows the curve of the coal fluid loss rate varying with time when the curing particles are respectively placed at position 3, position 4, position 5, or at positions 4 and 5 simultaneously.

From Fig 8, it can be seen that during the movement and temporary plugging of the temporary plugging particles in the coal fractures, the fluid loss rate initially increases gradually with the injection of the inlet fluid. When the fluid fills the internal fractures of the coal, the fluid loss rate reaches its maximum value and remains relatively stable before the particles are fully sealed. At each temporary plugging position, the stable value of the fluid loss rate is basically the same. At an injection pressure of 8 MPa, the stable value is approximately 0.162 cm³/s.

When the temporary plugging particles approach the crack network temporary plugging position, the fluid loss rate gradually decreases until the particles are stably sealed, and the fluid loss rate becomes stable. Moreover, the fluid loss rate varies when the particles are sealed at different temporary plugging positions. When the particles are sealed at position 5, the stable value of the fluid loss rate is the largest, at 0.152 cm³/s; when sealed at position 4, the fluid loss rate is 0.137 cm³/s; when sealed at position 1, the fluid loss rate is 0.131 cm³/s; when sealed at position 2, the fluid loss rate is 0.115 cm³/s; when sealed at positions 4 and 5 simultaneously, the fluid loss rate is 0.095 cm³/s; when sealed at position 3, the stable value of the fluid loss rate is 0.086 cm³/s; and when sealed at positions 1 and 2 simultaneously, the fluid loss rate is 0.037 cm³/s.

The numerical simulation results indicate that the temporary plugging position of the solidification plug affects the fluid loss rate of the coal after the plug completes its temporary plugging. By comparing the stable values of fluid loss rates after temporary plugging at different positions, it can be found that for temporary plugging positions close to the inlet, when the plug is sealed at a single position, the stable value of the fluid loss rate after temporary plugging is greater than that when the particles are sealed at multiple positions. For example, when the plug is sealed at position 1 and position 2 respectively, the stable value of the fluid loss rate after temporary plugging is greater than that when the plug is sealed simultaneously at position 1 and position 2; when the plug is sealed at position 4 and position 5 respectively, the stable value of the fluid loss rate after temporary plugging is greater than that when the plug is sealed simultaneously at position 4 and position 5.

Moreover, based on the stable fluid loss rates after temporary plugging, position 5 > position 4 > position 1 > position 2, and based on the stable fluid loss rates after temporary plugging, simultaneous temporary plugging at position 4 and position 5 > temporary plugging at position 3 > simultaneous temporary plugging at position 1 and position 2. This indicates that the closer the temporary plugging position is to the inlet, the smaller the stable value of the fluid loss rate after temporary plugging. This shows that in the actual pressure injection process for temporary plugging, the closer the temporary plugging position is to the inlet, the smaller the fluid loss rate after temporary plugging.

(4) The variation law of fluid loss rate under different injection pressures

**Fig 9:**
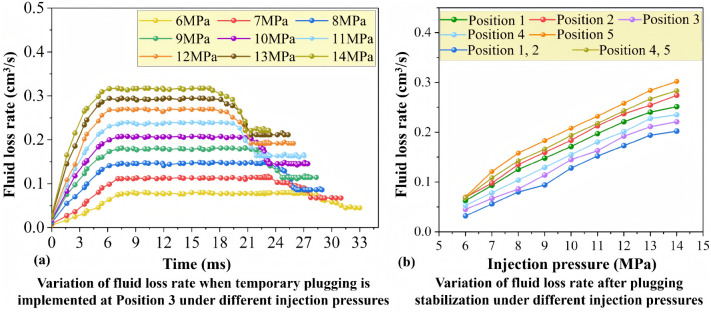
Fluid loss rate during temporary plugging at position 3 under different injection pressures.

Due to the influence of injection pressure on the shut – off fracturing fluid loss rate, an injection pressure range of 2–10 MPa was set. Position 3 of the temporary plugging location was selected as the research object, and the curve of fluid loss rate over time was obtained, as shown in Fig 9(a).

Based on the numerical simulation results, within the injection pressure range of 2–10 MPa, during the movement of the temporary plugging particles and the temporary plugging process of the coal, the fluid loss rate initially increased with the injection of the inlet fluid. When the injected fluid filled the internal pores and fractures of the coal, the fluid loss rate reached its maximum value and remained relatively stable before the particles were sealed. As the injection pressure increased, the stable value gradually increased, from approximately 0.079 cm³/s at an injection pressure of 6 MPa to approximately 0.318 cm³/s at an injection pressure of 14 MPa.

In addition, the time variation of the fluid loss rate also changes with the injection pressure, that is, the time for the particle temporary plugging to be completed varies. From the numerical simulation results, as the injection pressure increases, the time for the temporary plugging particles to complete the temporary plugging gradually decreases, from approximately 124.6 minutes at an injection pressure of 6 MPa to approximately 87.7 minutes at an injection pressure of 14 MPa. When the temporary plugging particles are sealed, the fluid loss rate increases from approximately 0.045 cm³/s at an injection pressure of 6 MPa to approximately 0.221 cm³/s at an injection pressure of 14 MPa.

When the temporary plugging particles are blocked at different positions, the curve of fluid loss rate of the model fluid under different injection pressures is shown in Fig 9(b). As can be seen from the figure, when the particles are blocked at a certain position, the greater the injection pressure, the greater the fluid loss rate after the particles are blocked. Under the same injection pressure, the fluid loss rate after blocking increases as the distance between the temporary plugging position and the injection inlet increases.

To study the influence of temporary plugging particle temporary plugging on the internal pore-fracture pressure and fluid loss rate of the coal, the basic dimension quantities selected are the fracture width, the distance between the temporary plugging position and the inlet, the injection pressure, the average internal pore-fracture pressure of the coal after temporary plugging, and the fluid loss rate at the coal outlet after temporary plugging. Based on the numerical simulation results, a dimensionless relationship formula is established. The obtained fluid loss rate data is nonlinearly fitted using the least square method to perform multivariate function fitting with the fracture width, the distance between the temporary plugging position and the inlet, the injection pressure, and the average internal pore-fracture pressure of the coal after temporary plugging. The obtained fitting formula is:


$$\begin{array}{c} v_{fl}=\frac{0.18{P_{ave}}^{\text{2}}-2.89P_{ave}+11.17P_{ent}+0.54\sqrt{d_{in}}+5.71}{0.21\sqrt{D_{eq}}+0.56} \end{array}$$ (7)

where $v_{fl}$ represents the fluid loss rate of the coal after the temporary temporary plugging particles achieve stable temporary plugging, cm³/s; $P_{ave}$ represents the average internal pressure of the coal after the temporary temporary plugging particles achieve stable temporary plugging, MPa; $P_{ent}$ represents the injection pressure, MPa; $d_{in}$ represents the distance from the temporary plugging position to the inlet, μm; $D_{eq}$ represents the equivalent width of the fractures at the temporary plugging position, μm.

**Fig 10:**
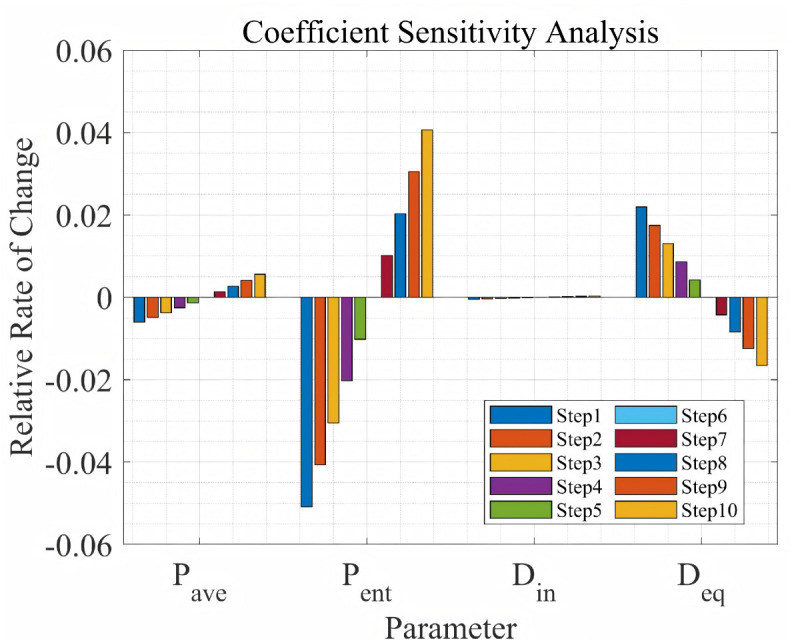
Sensitivity analysis of relevant parameters.

Related parameters were selected for the sensitivity analysis, as illustrated in Fig 10. Steps 1–10 represent the ten selected data groups, each corresponding to different step lengths, reflecting the changes of each parameter at various values. The fluid loss rate decreases with the equivalent width of the fractures at the temporary plugging position, while it increases with increases in the injection pressure and distance from the temporary plugging position to the inlet. Moreover, the differences in the relative rate of change under different step lengths in Fig 10 indicate that the influence of crack width and temporary plugging position on fluid loss during temporary plugging fracturing is relatively small. The injection pressure has a significant impact on the fluid loss rate. Meanwhile, under the same conditions of crack width, temporary plugging position, and injection pressure, there is a quadratic function relationship between the temporary plugging pressure and the fluid loss rate. The fitting formula R^2^ is 0.998. The numerical simulation calculation shows a good fit between the actual values and the predicted values of the fitting formula.

### 5.3. Verification of the temporary plugging particle’s effect on pressure increase and fluid loss patterns

(1) SC-CO_2_ - Pressure-time variation pattern during the hot alkali injection process

The solidified particle temporary plugging and pressure boosting law mainly refers to the pressure variation law of the fluid in the connected pores and fractures within the coal over time. Due to the limitations of the test equipment and test conditions, the fluid pressure in the coal pore fractures is characterized by the injection pressure and the pressure gauge at the fluid inlet of the clamping device.

**Fig 11:**
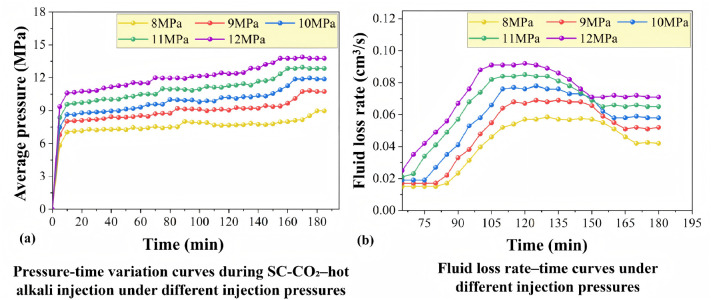
Changes in pressure and fluid loss rate under different injection pressures.

During the experiment, the injection pressure, the pressure of the clamping device, and the inlet pressure changes were monitored under different injection pressures when injecting SC-CO_2_, injecting an alkaline solution, and after the formation of solidified particles. The pressure-time variation curves were plotted, as shown in Fig 11(a).

From the pressure-time curves of the SC-CO_2_-thermal alkali injection experiment, it can be found that the pressure variation law with time under different injection pressures has certain similarities.

During the SC-CO_2_ injection stage, due to the pressure relief effect of the cavity in the injection pipe and the internal pore fractures of the coal sample, the pressure at the injection inlet is less than the injection pressure provided by the injection pump during the process of SC-CO_2_ moving from the injection inlet to the coal sample’s pore fractures. In the initial stage of SC-CO_2_ injection, SC-CO_2_ fills the cavity of the injection inlet and begins to diffuse into the coal sample’s pore fractures within a short period of time. The pressure at the injection inlet increases rapidly during this stage. With the continuous injection of SC-CO_2_, the pressure inside the coal sample’s pore fractures and at the injection inlet continuously increases. Due to the strong diffusion ability of SC-CO_2_, the pressure at the injection inlet increases to be basically equal to the injection pressure after about 1 hour of injection. In addition, the greater the injection pressure, the shorter the time it takes for the pressure at the injection inlet to reach the injection pressure.

When the pressure at the injection inlet increases to be basically equal to the injection pressure and remains stable, the injection of SC-CO_2_ is stopped, the remaining SC-CO_2_ in the pipeline is emptied, and then the injection of alkaline solution begins. Considering the compressibility of SC-CO_2_, the injection pressure of the alkaline solution is higher than that of the injection of SC-CO_2_.

In the initial stage of alkaline solution injection, the SC-CO_2_ in the coal sample and the contacting alkaline solution rapidly undergo a chemical reaction, generating solidified particles and water. Due to the influence of the chemical reaction, the pressure inside the coal sample and at the injection inlet decreases. With the injection of alkaline solution, the pressure inside the coal sample and at the injection inlet gradually increases. Due to the fact that there are fewer solidified particles generated in the early stage of the chemical reaction and the SC-CO_2_ continuously undergoing chemical reactions with the contacting alkaline solution, the increase in pressure inside the coal sample and at the injection inlet is slow. With the continuous injection of alkaline solution, the number of temporary plugging particles gradually increases, and as the fluid flows, the temporary plugging particles move and accumulate in the connected pore fractures of the coal, thereby causing the pressure inside the coal sample and at the injection inlet to increase rapidly, achieving the effect of temporary plugging and pressure boosting by solidified particles. When the solidified particles form a stable accumulation, the pressure inside the coal sample and at the injection inlet remains stable.

(2) SC-CO_2_ - Variation law of fluid loss rate over time during the process of thermal alkali injection

During the process of temporarily blocking the injection of fracturing fluid, the water leakage from the coal is the main cause of a significant decrease in water pressure. The temporary plugging particles’ temporary plugging of the coal fracture network can reduce fluid loss and significantly enhance the hydraulic enhancement of permeability of the coal. In the SC-CO_2_ - hot alkali injection experiment, due to the limitations of the experimental equipment and conditions, it was impossible to directly monitor the fluid loss rate of the coal sample. By referring to the fluid loss rate formula, during the experiment, the flow meter readings at the inlet and outlet of the coal sample holder at different times were recorded respectively, and the fluid loss rate was calculated.


$$\begin{array}{c} v_{l}=\frac{d_{v}}{Ad_{t}} \end{array}$$ (8)

where $v_{l}$ represents the fluid loss rate, cm³/s; $d_{v}$ is the change in fluid volume; $d_{t}$ is the change in time; A is the cross-sectional area of the coal sample, cm².

During the SC-CO_2_ injection process, there is no blocking by temporary particles or fluid loss due to moisture. Therefore, the focus is on monitoring the changes in liquid flow velocity at the inlet and outlet of the holder after the injection of the alkaline solution begins. The fluid loss rate is calculated. Curves depicting the variation of the fluid loss rate with time under different injection pressures are plotted, as shown in Fig 11(b):

From the fluid loss rate-time curves under different injection pressures during the SC-CO_2_ - hot alkaline injection experiment, it can be observed that the variation patterns of the fluid loss rate with time under different injection pressures have certain similarities.

During the initial stage of the alkaline solution injection, the SC-CO_2_ within the coal sample and the contacting alkaline solution rapidly undergo chemical reactions, generating solidified particles and water. As the fluid is injected and transported, the fluid loss rate gradually increases. When the fluid fills the internal pores and fractures of the coal, the fluid loss rate reaches its maximum value and remains relatively stable before the particles block the coal. As the solidified particles are generated and transported within the coal and accumulate, the fluid loss rate of the coal sample gradually decreases. When the alkaline solution completely reacts with the initially injected SC-CO_2_ within the coal sample and the stable blocking particles are formed to seal the pores and fractures of the coal, the fluid loss rate of the coal sample remains relatively stable.

(3) Analysis of the pressure-enhancement law for solidified particle temporary plugging

**Fig 12:**
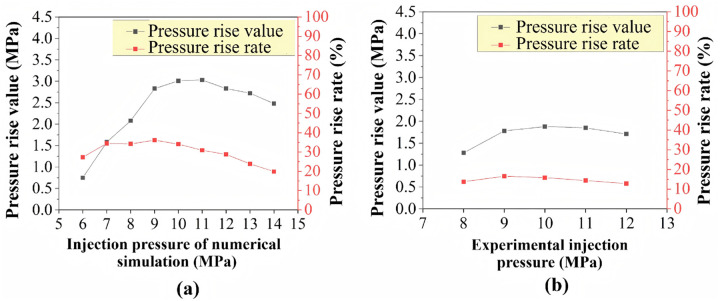
The pressure value and pressure increase rate for the solidified particle temporary plugging.

The fluid pressure in the coal fractures obtained from the experiment was compared with the pressure rise law of the temporary plugging particles within the connected fractures obtained through numerical simulation in Section 5.1. Due to the limitations of the experimental conditions, the maximum pressure inside the coal sample could not be obtained. Therefore, the main comparison was made between the pressure-time variation curves obtained by the injection inlet pressure and the curves showing the variation of the average pressure inside the fractures of the connected pores with time under different injection pressures in Fig 5 of Section 5.1. Since the numerical simulation is based on the fluid-solid coupling method and simulates the movement, accumulation, and temporary plugging process of particles under the action of fluid and coal, the main process is the transport, accumulation, and pressure rise process of the solidified particles formed after the reaction between SC-CO_2_ and alkaline solution. Through the comparison, it was found that under the condition that the fluid in both the experiment and the numerical simulation was injected at a constant pressure, due to the temporary plugging effect of the solidified particles blocking the connected fractures of the coal, the pressure inside the coal gradually increased in both the experimental and numerical simulation results. The temporary plugging particle temporary plugging effect caused an increase in the pressure inside the fractures. To further verify the consistency between the experiment and the numerical simulation, the pressure rise values and pressure rise rates of the solidified particles temporarily blocking the temporary plugging were calculated respectively, and curves were drawn, as shown in Fig 12. Here, the pressure rise value is the pressure increase value caused by the particle temporary plugging; the pressure rise rate is the percentage of the pressure increase caused by the particle temporary plugging.

Fig 12(a) shows the pressure increase values and rates under injection pressures ranging from 6 MPa to 14 MPa in the numerical simulation results. Fig 12(b) presents the pressure increase values and rates under injection pressures ranging from 8 MPa to 12 MPa in the experimental results.

In Fig 12(a), when the injection pressure is 6 MPa, the minimum pressure increase value for particle temporary plugging is 0.75 MPa. As the pressure increases, the pressure increase value gradually increases. When the injection pressure is 9 MPa, the pressure increase value reaches the maximum of 3.23 MPa. After that, as the injection pressure increases, the pressure increase value decreases slowly. When the injection pressure is 6 MPa, the pressure increase rate for particle temporary plugging is 27.27%. When the injection pressure is 9 MPa, the pressure increase rate for particle temporary plugging reaches the maximum of 34.49%, and then the pressure increase rate decreases slowly. In Fig 12(b), when the injection pressure is 8 MPa, the minimum pressure increase value for particle temporary plugging is 1.18 MPa. As the pressure increases, the pressure increase value gradually increases. When the injection pressure is 10 MPa, the pressure increase value reaches the maximum of 1.88 MPa. When the injection pressure is 11 MPa, the pressure increase value decreases to 1.85 MPa. When the injection pressure is 12 MPa, the pressure increase value continues to decrease slowly to 1.77 MPa. When the injection pressure is 8 MPa, the pressure increase rate for particle temporary plugging is 12.85%. When the injection pressure is 9 MPa, the pressure increase rate for particle temporary plugging reaches the maximum of 16.51%, and then the pressure increase rate decreases slowly. After that, the pressure increase rate decreases slowly.

Under the injection pressure range of 8 MPa to 12 MPa, the pressure increase values and rates in the numerical simulation results of Fig 12(a) and the experimental results of Fig 12(b) both show a trend of increasing first and then decreasing.

(4) Analysis of the temporary plugging particle’s effect on fluid loss regulation

The variation pattern of fluid loss rate over time obtained from the experiment was compared with that of the fluid loss rate over time obtained through numerical simulation in Section 5.2. Considering the injection and chemical reaction process of the SC-CO_2_ - thermal alkali injection experiment, the main comparison was made between the change in fluid loss rate after the start of alkali injection and the time-varying curves of fluid loss rate under different injection pressures in Fig 9 of Section 5.2. Through the comparison, it was found that the fluid loss rates in both the numerical simulation and the experimental results increased first to a certain value, then gradually decreased due to the blocking effect of the temporary plugging particles, and remained stable after the blocking was completed. The blocking effect of the temporary plugging particles caused the reduction in fluid loss rate.

**Fig 13:**
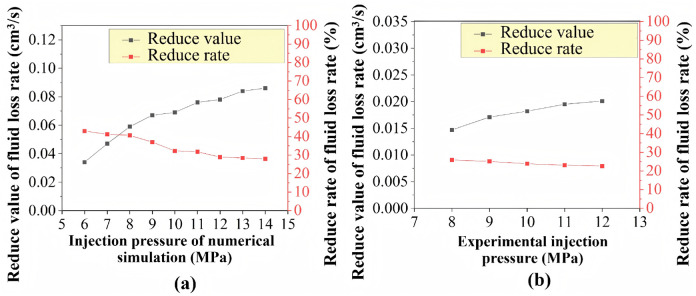
The pressure value and pressure increase rate for the solidified particle temporary plugging.

The reduction values and reduction rates of fluid loss rate due to the temporary plugging particles were calculated separately for the numerical simulation and the experiment, and curves were plotted, as shown in Fig 13. The reduction value of fluid loss rate is the reduction in fluid loss rate of the coal caused by the temporary plugging particles; the reduction rate of fluid loss rate is the percentage reduction in fluid loss rate caused by the temporary plugging particles.

Fig 13(a) presents the fluid loss rate, the reduction value of fluid loss rate, and the reduction rate of fluid loss rate under an injection pressure of 6–14 MPa in the numerical simulation results. Fig 13(b) shows the reduction value of fluid loss rate and the reduction rate of fluid velocity under an injection pressure of 8–10 MPa in the experimental results.

In Fig 13(a), when the injection pressure is 6 MPa, the outlet fluid velocity under the particle temporary plugging effect is 0.045 cm³/s. As the pressure increases, the reduction value of the fluid loss rate gradually increases. When the injection pressure is 14 MPa, the outlet fluid velocity is 0.221 cm³/s.

When the injection pressure is 6 MPa, the reduction value of the fluid loss rate under particle temporary plugging is the smallest, at 0.034 cm³/s. As the pressure increases, the reduction value of the fluid loss rate increases slowly. When the injection pressure is 14 MPa, the reduction value of the fluid velocity is 0.086 cm³/s.

When the injection pressure is 6 MPa, the reduction rate of the fluid loss rate under particle temporary plugging is 43.03%. As the pressure increases, the reduction rate of the fluid loss rate gradually decreases. When the injection pressure is 14 MPa, the reduction rate of the fluid velocity is 28.01%.

The numerical simulation results are consistent with the curves of the reduction value of fluid loss rate and the reduction rate of fluid velocity in Fig 13(b) of the experimental results.

Based on comprehensive analysis, the numerical simulation results show that the pressure increase value increased from 0.75 MPa to a peak of 3.23 MPa, and then fell back to 2.45 MPa, with an overall range of 0.75 to 3.23 MPa. The peak pressure increase effect is significant. The experimental results showed that the pressure increase value increased from 1.18 MPa to a peak of 1.88 MPa, and then fell back to 1.77 MPa, with an overall range of 1.18–1.88 MPa. The experimental peak value was about 63% of the simulated peak value. The decrease in filtration rate in the numerical simulation results increased significantly from 0.034 cm^3^/s to 0.086 cm^3^/s, with a smooth curve and no obvious fluctuations. The decrease in fluid loss rate in the experimental results increased from 0.015 cm^3^/s to 0.020 cm^3^/s, and the curve was also smooth, but the increase was lower than the numerical simulation results, ranging from 16% to 23% of the simulated value.

There is a certain deviation between numerical simulation and experimental results. Based on the experimental results, the reason is that the network fluid equation did not consider the fracture structure and injection parameters, and did not take into account the dynamic attenuation of permeability caused by temporary plugging particles during the fluid loss process. The evolution of porosity and particles migration are not correlated with fluid loss rate, injection parameter pressure, and fracture structure parameters. The particle velocity did not take into account the drag force and crack width of the fluid loss flow field.

Meanwhile the values of particle temporary plugging pressure increase, pressure increase rate, reduction value of fluid loss rate, and reduction rate of fluid loss rate obtained through numerical simulation are consistent with the experimental results: as the injection pressure increases, both the temporary plugging pressure increase value and the pressure increase rate show a trend of increasing first and then decreasing. The outlet flow rate gradually increases, the reduction value of the fluid loss rate slowly increases, and the reduction rate of the fluid loss rate gradually decreases.

To analyze the reasons: in coal temporary plugging fracturing, there is a significant dynamic coupling relationship between the injection pressure and the filtration rate of the fluid. The filtration rate is usually proportional to the pressure difference inside and outside the coal sample, and at the same time, the filtration rate directly affects the internal pressure of the coal sample. Fluid loss leads to a decrease in the internal pressure of the coal sample. Temporary plugging fracturing reduces filtration by temporary plugging the matrix pores or natural fractures. In the initial stage of fracturing without temporary plugging, the injection pressure increases slowly, and the filtration rate is high. After the temporary plugging agent is used for plugging, the filtration rate decreases, the internal pressure of the coal sample rises, and the pressure curve presents a “plateau” or “stepwise climbing” pattern. The injection pressure further affects the filtration behavior by changing the flow resistance of the fluid and the pressure inside the fractures. If the injection pressure is too high, it will exacerbate fluid loss. Therefore, in the numerical simulation and experiments, when the injection pressure increases to a certain value, the temporary plugging pressure increase value and the pressure increase rate decrease. In the actual temporary plugging fracturing process, it is necessary to reasonably set the injection parameters of temporary plugging fracturing, control the filtration rate at the same time, maintain a reasonable injection pressure, and achieve precise control.

## 6. Conclusion

(1) The distance between the temporary plugging position of particles and the injection port, as well as the number of blocked fractures, affect the pressure distribution, average pressure, fluid flow distribution, and fluid loss rate of the coal fracture network. When particles are blocked at different positions, there are differences in the average pressure of the fracture network, with a maximum of 12.03 MPa and a minimum of 8.05 MPa. For temporary plugging positions close to the entrance, when the particles are blocked in a single position, the average pressure after blocking is lower than when the particles are blocked in multiple positions. The closer the temporary plugging position of the particles is to the injection port, the lower the average pressure, and the farther the temporary plugging position is from the injection port, the better the average boosting effect of temporary plugging fracturing. When particles are blocked at different positions, there are differences in fluid loss rate, with a maximum of 0.152 cm^3^/s and a minimum of 0.037 cm^3^/s. The closer the particle temporary plugging position is to the inlet, the lower the fluid loss rate after blockage. For temporary plugging position close to the inlet, when the particle is temporary plugging at a single position, the fluid loss rate after blockage is greater than when the particle is blocked at multiple positions.
(2) The injection pressure affects the pressure boosting and fluid loss effect. As the injection pressure increases, the temporary plugging pressure increase value of particles increases from 0.75 MPa to 3.23 MPa and then slowly decreases; The temporary plugging pressure increase rate of particles increased from 27.27% to 34.49% and then slowly decreased. As the injection pressure increases, the pressure increase value and pressure increase rate in the coal fracture network temporary plugging by particles show a trend of first increasing and then decreasing. As the injection pressure increases, the decrease in fluid loss rate continues to increase from 0.034 cm^3^/s to 0.086 cm^3^/s; The reduction rate of fluid loss rate gradually decreased from 43.03% to 28.01%.
(3) Construct equations to describe the relationship between various parameters and fluid loss rate. Injection pressure has a significant impact on fluid loss rate, while fracture width and temporary plugging position have relatively small effects on fluid loss rate during temporary plugging fracturing. The fitting formula R^2^ is 0.988.
(4) Pressure difference is the driving force behind fluid flow. The flow velocity of the fluid in the coal fracture network depends on the pressure difference of the coal fracture network, and the different positions of the pores result in differences in the flow field of the fluid inside the pores.
(5) The particle temporary plugging pressure increase values, pressure increase rates, reduction values of the outlet volumetric flow rate, and reduction rates of the outlet volumetric flow rate obtained from the experimental results are consistent with the numerical model results.
(6) This study can provide scientific basis and data support for the application of temporary plugging and fracturing technology in coal to increase the extraction of coal seam gas, and to address the issues of particle transport and temporary plugging pressure control.
