# Supplementary material for: Numerical simulation study on pressure response and fluid loss mechanism of coal fracture network under particle temporary plugging
Source: PLoS One. 2026 Jul 24;21(7):e0352681. doi: 10.1371/journal.pone.0352681 (PMC13399314; doi:10.1371/journal.pone.0352681)
Supplement: Attachment — Submitted filename: Response to Reviewers.doc [file pone.0352681.s001.doc]

**Dear editors and reviewers:**

**We would like to express our gratitude to the esteemed experts for your valuable feedback. We have diligently revised the manuscript in accordance with the suggestions provided, and we hope that the paper will be accepted. Additionally, we extend our sincere appreciation to the editors and reviewers for your contributions to the advancement of scientific research !**

Yours sincerely

**Doctor Chen Jian**

**July 1, 2026**

# **Explanations about Modifications**

**Reviewer 1#：**

**Question1**. This manuscript does not mention a sensitivity analysis of the model with respect to different input parameters.

**Answer:** We appreciate the expert feedback. An analysis of the influences of relevant parameters on the model has been added.

$$v_{fl}=\frac{0.18P_{ave}^{2}-2.89P_{ave}+11.17P_{ent}+0.54\sqrt{d_{in}}+5.71}{0.21\sqrt{D_{eq}}+0.56}$$

Where $v_{fl}$represents the fluid loss rate of the coal body after the temporary temporary plugging particles achieve stable temporary plugging, cm³/s; $P_{ave}$ represents the average internal pressure of the coal body after the temporary temporary plugging particles achieve stable temporary plugging, MPa; $P_{ent}$ represents the injection pressure, MPa; $d_{in}$represents the distance from the temporary plugging position to the inlet, μm; $D_{eq}$ represents the equivalent width of the fractures at the temporary plugging position, μm.

The modified content (Line 1-8, Page 34) is as follows:

Related parameters were selected for the sensitivity analysis, as illustrated in **Fig. 1** (**Fig. 10** in the paper, Line 1, Page 34). Steps 1 to 10 represent the ten selected data groups, each corresponding to different step lengths, reflecting the changes of each parameter at various values. The fluid loss rate decreases with the equivalent width of the fractures at the temporary plugging position, while it increases with increases in the injection pressure and distance from the temporary plugging position to the inlet. Moreover, the differences in the relative rate of change under different step lengths in **Fig. 1** indicate that the influence of crack width and temporary plugging position on fluid loss during temporary plugging fracturing is relatively small. The injection pressure has a significant impact on the fluid loss rate. Meanwhile, under the same conditions of crack width, temporary plugging position, and injection pressure, there is a quadratic function relationship between the temporary plugging pressure and the fluid loss rate. The fitting formula R^2^ is 0.998. The numerical simulation calculation shows a good fit between the actual values and the predicted values of the fitting formula.

**Fig. 1.** Sensitivity analysis of relevant parameters

**Question2**. The conclusion does not fully capture the significance of the model or the experimental findings. Consider revising it to better reflect the novelty and contributions of the work, as well as including key quantitative results.

**Answer:** Based on expert feedback, the conclusion has been revised to include descriptions of quantitative results (Lines 5-21, Page 45; Lines 1-18, Page 46).

The modified conclusion is as follows:

**Conclusion:**

(1) The distance between the temporary plugging position of particles and the injection port, as well as the number of blocked fractures, affect the pressure distribution, average pressure, fluid flow distribution, and fluid loss rate of the coal fracture network. When particles are blocked at different positions, there are differences in the average pressure of the fracture network, with a maximum of 12.03 MPa and a minimum of 8.05 MPa. For temporary plugging positions close to the entrance, when the particles are blocked in a single position, the average pressure after blocking is lower than when the particles are blocked in multiple positions. The closer the temporary plugging position of the particles is to the injection port, the lower the average pressure, and the farther the temporary plugging position is from the injection port, the better the average boosting effect of temporary plugging fracturing. When particles are blocked at different positions, there are differences in fluid loss rate, with a maximum of 0.152 cm3/s and a minimum of 0.037 cm3/s. The closer the particle temporary plugging position is to the inlet, the lower the fluid loss rate after blockage. For temporary plugging position close to the inlet, when the particle is temporary plugging at a single position, the fluid loss rate after blockage is greater than when the particle is blocked at multiple positions.

(2) The injection pressure affects the pressure boosting and fluid loss effect. As the injection pressure increases, the temporary plugging pressure increase value of particles increases from 0.75 MPa to 3.23 MPa and then slowly decreases; The temporary plugging pressure increase rate of particles increased from 27.27% to 34.49% and then slowly decreased. As the injection pressure increases, the pressure increase value and pressure increase rate in the coal fracture network temporary plugging by particles show a trend of first increasing and then decreasing. As the injection pressure increases, the decrease in fluid loss rate continues to increase from 0.034 cm3/s to 0.086 cm3/s; The reduction rate of fluid loss rate gradually decreased from 43.03% to 28.01%.

(3) Construct equations to describe the relationship between various parameters and fluid loss rate. Injection pressure has a significant impact on fluid loss rate, while fracture width and temporary plugging position have relatively small effects on fluid loss rate during temporary plugging fracturing. The fitting formula R2 is 0.988.

(4) Pressure difference is the driving force behind fluid flow. The flow velocity of the fluid in the coal fracture network depends on the pressure difference of the coal fracture network, and the different positions of the pores result in differences in the flow field of the fluid inside the pores.

(5) The particle temporary plugging pressure increase values, pressure increase rates, reduction values of the outlet volumetric flow rate, and reduction rates of the outlet volumetric flow rate obtained from the experimental results are consistent with the numerical model results.

(6) This study can provide scientific basis and data support for the application of temporary plugging and fracturing technology in coal to increase the extraction of coal seam gas, and to address the issues of particle transport and temporary plugging pressure control.

**Question3.** Several similar but inconsistent expressions are used throughout the manuscript, including "temporary plugging ," "temporarily blocking," and "temporary blockage." In some cases, multiple expressions are used interchangeably even within the same context. The authors are advised to standardize the key terminology throughout the paper, especially in the title, abstract, methods, results, figure captions, and conclusions.

**Answer:** Thank you for the expert's revision suggestions. We have unified the key terms in the text. For example, "temporary plugging", "temporarily plugging", and "temporary blockage" have been unified as "temporary plugging".

**Question4.** The English expression and academic writing specifications of the whole text still need to be systematically polished. There are some language and formatting problems in the current manuscript, such as long titles or improper collocations in some sentences. It is recommended that authors polish the title, abstract, introduction, conclusion and diagram description in English during the revision process, and unify the terminology, symbols and expression style.

**Answer:** We appreciate the expert feedback.The expressions in the paper have been revised in accordance with expert opinions, as demonstrated in **Fig. 2**：

**Fig.2.** Proof about proofreading

**Question5.** The manuscript does not explain how the segmentation threshold was determined. Since these steps directly affect the realism of the geometric model, the authors should provide a more detailed description of the image-processing workflow, parameter selection criteria, and representativeness verification.

**Answer:** Based on the expert's revision suggestions, additional explanations are provided for the CT scan image processing process. The modified content (Line 7-18, Page 7; Line 1-2, Page 8) is as follows:

The CT scanning experiment obtained a two-dimensional CT grayscale image of the coal sample, which was imported into Avizo for processing. Image processing techniques such as filtering, noise reduction, and threshold segmentation were applied to obtain a binary image.

The ultimate goal of noise reduction is to provide high signal-to-noise ratio data for coal and rock fracture network modeling, while avoiding the loss of microstructural information due to excessive smoothing. Selecting a median filter to achieve image denoising not only removes noise from the image but also preserves the edge features of the image, resulting in good denoising and image restoration effects. Based on the bimodal distribution characteristics of the two-phase grayscale histogram of coal rock matrix and pores, a globally optimal bimodal adaptive threshold iteration algorithm is employed, combined with cross-checking of multiple slices within the same batch, to determine the segmentation threshold. Avoid the upper and lower end faces of the coal sample, the edge processing disturbance zone, and the local abnormal high-density mineral inclusion zone. Select typical cross sections from the upper, middle, and lower layers at equal intervals along the axial direction of the coal sample. Images with obvious fracture structures were selected for processing, and the final geometric structure of the micro-macroscopic connected pores and fractures of coal was obtained, as shown in **Fig. 3** (**Fig. 1** in the paper, Line 5, Page 8).

**Fig. 3.** Geometric Model Construction Process

**Reviewer 2#：**

**Question1.** The research gap in the abstract is not sufficiently clear and should be further refined. The authors are encouraged to revise the abstract following a more logical structure, including background, research gap, methodology, key results, and significance. In addition, the key findings should be highlighted more clearly and, where possible, supported by quantitative results. The current abstract contains useful information, but it is relatively long and would benefit from a more concise and focused presentation.

**Answer:** Thank you for the expert's revision suggestions. Based on expert feedback, the abstract has been revised to include descriptions of quantitative results (Lines 1-22, Page 2).

The modified abstract is as follows:

**Abstract:** To address the issue of fluid loss leading to pressure failure when applying temporary plugging fracturing technology from the oil industry to enhance gas recovery in coal mines, and considering the current low efficiency of CO_2_ solidification and utilization, a new method is proposed to inject SC-CO_2_ and hot alkali solution together into coal to produce solidified particles as temporary plugging agents for solidification and utilization of CO_2_ and fracturing transformation of coal. At present, the mechanisms governing pressure increase during temporary plugging with solidified CO_2_ particles, as well as the relationship between internal pore and fracture pressures after plugging and fluid loss rate, remain unclear. Based on CT images, a complex geometric model of interconnected pores and fractures of coal is constructed. The morphology of solidified CO_2_ particles is obtained via microscopic scanning, and conducts numerical simulation research on fluid solid coupling. Clearly define the pressure and flow distribution inside the coal under different temporary plugging positions and injection conditions. Revealing the pressure and fluid loss law inside the coal after temporary plugging. The results show that when particles are temporary plugging at different positions, the average internal pressure in the coal ranges from 8.05 MPa to 12.03 MPa. The fluid loss rate varies from 0.037 cm^3^/s to 0.152 cm^3^/s. Establish equations for the relationship between injection pressure, fracture size, temporary plugging location, internal pressure, and fluid loss rate, with a fitting formula R^2^ of 0.988. Furthermore, an experimental setup and methodology involving supercritical CO_2_ and hot alkali liquor injection were designed to validate the accuracy of the numerical simulation results regarding pressure enhancement and fluid loss under solidified particle plugging. This study provides theoretical support for enhanced coalbed methane recovery and the development of integrated technologies for underground CO_2_ storage and hydraulic control of gas emissions.

**Question2.** Some keywords may not be suitable for indexing the paper. For example, “connectivity of pores and fractures” and “temporary plugging with pressure increase” appear more like descriptive phrases than effective keywords. The authors should revise the keywords to better reflect the main topic, methods, materials, and application of the study. Potential keywords may include terms related to temporary plugging fracturing, supercritical CO_2_, coalbed methane, fluid loss, pore–fracture structure, and coupled fluid–solid simulation.

**Answer:** Based on expert feedback, the keywords (Lines 1-2, Page 3) has been modified as follows:

**Keywords:** Temporary plugging fracturing; Interconnected pore and fracture; Pressure response; Fluid loss rate.

**Question3.** The significance of the study should be further highlighted at the end of the Introduction. The authors should clearly explain how this work advances the understanding of temporary plugging mechanisms, pressure enhancement, and fluid loss behaviour in coal pores and fractures. The practical relevance to coalbed methane recovery, CO_2_ storage, and gas emission control should also be emphasised.

**Answer:** Thank you for the expert's revision suggestions. The introduction has been modified as follows:

(Lines 4-7, Page 3)

Coalbed methane is a coal related energy source with abundant resources. Coal reservoirs generally have characteristics such as low permeability, high adsorption, and strong heterogeneity. Traditional hydraulic fracturing technology faces many challenges in coal reservoir transformation and gas extraction.

(Lines 19-21, Page 3; Lines 1-5, Page 4)

The resource utilization and storage of CO_2_ has become an important path to achieve the “dual carbon” strategic goals. Supercritical CO_2_ (SC-CO_2_) has low viscosity, strong permeability, and good injection effect in coal seams, and shows unique advantages in fields such as coal seam gas displacement and coal reservoir fracturing modification. However, the existing SC-CO_2_ fracturing technology mainly focuses on its physical and mechanical effects as a fracturing medium, and pays insufficient attention to the solidification and utilization of CO_2_ after injection into coal rock. The efficiency of CO_2_ solidification and storage is low, and the potential of CO_2_ resource utilization in the fracturing process has not been fully exploited.

(Lines 13-15, Page 6)

This study can provide scientific basis and data support for the temporary plugging and pressure control issues faced by coal mines in applying temporary plugging and fracturing technology to increase coalbed methane production.

**Question4.** The numerical modelling methodology requires further clarification. The authors should clearly state which simulation software was used, how the numerical model was constructed from the CT-based pore–fracture structure, and what assumptions were adopted in the coupled fluid–solid simulation. In addition, the determination of key simulation parameters should be explained in more detail, including their sources, units, and physical meanings.

**Answer:** Thank you for the expert's revision suggestions. The numerical modelling methodology has been clarified as follows:

(Line 7-18, Page 7; Line 1-2, Page 8)

The CT scanning experiment obtained a two-dimensional CT grayscale image of the coal sample, which was imported into Avizo for processing. Image processing techniques such as filtering, noise reduction, and threshold segmentation were applied to obtain a binary image.

The ultimate goal of noise reduction is to provide high signal-to-noise ratio data for coal and rock fracture network modeling, while avoiding the loss of microstructural information due to excessive smoothing. Selecting a median filter to achieve image denoising not only removes noise from the image but also preserves the edge features of the image, resulting in good denoising and image restoration effects. Based on the bimodal distribution characteristics of the two-phase grayscale histogram of coal rock matrix and pores, a globally optimal bimodal adaptive threshold iteration algorithm is employed, combined with cross-checking of multiple slices within the same batch, to determine the segmentation threshold. Avoid the upper and lower end faces of the coal sample, the edge processing disturbance zone, and the local abnormal high-density mineral inclusion zone. Select typical cross sections from the upper, middle, and lower layers at equal intervals along the axial direction of the coal sample.

(Lines 5-15, Page 9)

Particle plugging hydraulic fracturing is a complex multi-field coupled process. The following basic assumptions are adopted in the numerical simulation: temporary plugging particles are assumed to be homogeneous, square elastic particles.

(1) The fluid is assumed to be Newtonian and incompressible.

(2) Temporary plugging fracturing involves multiple processes such as particle migration, pressure maintenance, and fracture initiation. Particle migration has been investigated in the paper “Migration of CO_2_-solidified temporary plugging particles used for temporary plugging fracturing in microscopic interconnected pores and fractures of coal”. This study focuses on plugging mechanisms, and fracture initiation is not considered.

(3) The pre-stress, heat exchange and coal swelling are neglected, and coal is treated as a linear structural material.

(Lines 1-10, Page 14)

The settings of relevant parameters for the numerical simulation are shown in **Table 1**.(**Table 4** in the paper, Line 4, Page 13).

**Table 1.** Parameters of numerical simulation

| Parameter | Value |
| --- | --- |
| Hydrodynamic viscosity (Pa·s) | 1.01e-3 |
| Fluid density (kg/m^3^) | 1 |
| Particle density (kg/m^3^) | 2700 |
| Young's modulus of particle (GPa） | 2.5 |
| Poisson ratio of particle (1) | 0.3 |
| Young's modulus of coal (GPa) | 0.1 |
| Poisson ratio of coal (1) | 0.314 |
| Coal density (kg/m^3^) | 1400 |

The fluid uses pure water as the medium, referring to the physical property standards of water at room temperature and pressure, and combining with the subtle environmental differences of simulated working conditions, the fluid dynamic viscosity is determined to be 1.01e-3 Pa·s, and the fluid density is 1kg/m^3^. The density of CO_2_ solidified particles is 2700 kg/m^3^. Based on the in-situ coal bulk density test results, the overall density of coal with pore structure is determined to be 1400 kg/m^3^. Based on the results of uniaxial and triaxial mechanical tests on coal, as well as simulation experience of similar coal seams, combined with the deformation characteristics of primary coal in the study area, the Young's modulus of coal is determined to be 3.5 GPa, and the Poisson ratio of coal is taken as 0.314, which can accurately characterize the deformation law of coal and rock under stress and adapt to the solid fluid coupling simulation conditions of this study.

**Question5.** It is suggested that the authors separate the experimental equipment from the experimental procedure to improve the clarity of the methodology section. In particular, the self-developed SC-CO_2_–hot alkali injection test system should be introduced in greater detail, including its main components, operating conditions, measurement methods, and how it was used to validate the numerical simulation results.

**Answer:** We appreciate the expert feedback. An introduce of the experimental system and measurement methods (Lines 10-20, Page 15) have been added.

Remove excess gas or liquid from the pipeline before injection. First, pressurize the CO_2_, open the injection valve of the coal sample holder, and inject SC-CO_2_ into the interior of the coal sample. When the pressure at the injection port remains stable, close the SC-CO_2_ injection valve. Open the liquid outlet valve in front of the coal sample holder inlet and discharge the remaining CO_2_ in the pipeline. Inject alkaline solution into the coal sample according to the experimental pressure setting. After the solidified particles form a stable accumulation, the pressure inside the coal sample and injection port remains stable, and the injection of alkali solution is stopped. Inject and record the inlet pressure of the gripper and the readings of the flow meters at the inlet and outlet of the coal sample gripper during the SC-CO_2_ and alkali solution processes. At the end of the experiment, unload the pressure and take out the coal sample.

**Question6.** There are some differences between the numerical simulation results and the laboratory experimental results. The authors should provide a clearer explanation and justification for these differences. Possible reasons may include simplifications in the numerical model, uncertainty in pore–fracture reconstruction, differences between idealised simulation conditions and experimental conditions, measurement errors, or limitations in parameter determination.

**Answer:** Thank you for the valuable feedback. The explanation and justification for differences between the numerical simulation results and the laboratory experimental results have been added. The additional content (Lines 7-21, Page 43; Lines 1-2, Page 44) is as follows:

Based on comprehensive analysis, the numerical simulation results show that the pressure increase value increased from 0.75 MPa to a peak of 3.23 MPa, and then fell back to 2.45 MPa, with an overall range of 0.75 to 3.23 MPa. The peak pressure increase effect is significant. The experimental results showed that the pressure increase value increased from 1.18 MPa to a peak of 1.88 MPa, and then fell back to 1.77 MPa, with an overall range of 1.18-1.88 MPa. The experimental peak value was about 63% of the simulated peak value. The decrease in filtration rate in the numerical simulation results increased significantly from 0.034 cm3/s to 0.086 cm^3^/s, with a smooth curve and no obvious fluctuations. The decrease in fluid loss rate in the experimental results increased from 0.015 cm^3^/s to 0.020 cm^3^/s, and the curve was also smooth, but the increase was lower than the numerical simulation results, ranging from 16% to 23% of the simulated value.

There is a certain deviation between numerical simulation and experimental results. Based on the experimental results, the reason is that the network fluid equation did not consider the fracture structure and injection parameters, and did not take into account the dynamic attenuation of permeability caused by temporary plugging particles during the fluid loss process. The evolution of porosity and particles migration are not correlated with fluid loss rate, injection parameter pressure, and fracture structure parameters. The particle velocity did not take into account the drag force and crack width of the fluid loss flow field.

**Question7.** The conclusion should be revised to better highlight the key findings of the study. At present, the main contributions are not sufficiently emphasised. The authors should summarise the major findings regarding plugging location, fracture size, injection pressure, pressure enhancement, and fluid loss rate. The conclusion should also clearly state the scientific and practical implications of the work.

**Answer:** We appreciate the expert feedback. The conclusion has been revised to include descriptions of quantitative results (Lines 5-21, Page 45; Lines 1-18, Page 46).

The modified conclusion is as follows:

**Conclusion:**

(1) The distance between the temporary plugging position of particles and the injection port, as well as the number of blocked fractures, affect the pressure distribution, average pressure, fluid flow distribution, and fluid loss rate of the coal fracture network. When particles are blocked at different positions, there are differences in the average pressure of the fracture network, with a maximum of 12.03 MPa and a minimum of 8.05 MPa. For temporary plugging positions close to the entrance, when the particles are blocked in a single position, the average pressure after blocking is lower than when the particles are blocked in multiple positions. The closer the temporary plugging position of the particles is to the injection port, the lower the average pressure, and the farther the temporary plugging position is from the injection port, the better the average boosting effect of temporary plugging fracturing. When particles are blocked at different positions, there are differences in fluid loss rate, with a maximum of 0.152 cm^3^/s and a minimum of 0.037 cm^3^/s. The closer the particle temporary plugging position is to the inlet, the lower the fluid loss rate after blockage. For temporary plugging position close to the inlet, when the particle is temporary plugging at a single position, the fluid loss rate after blockage is greater than when the particle is blocked at multiple positions.

(2) The injection pressure affects the pressure boosting and fluid loss effect. As the injection pressure increases, the temporary plugging pressure increase value of particles increases from 0.75 MPa to 3.23 MPa and then slowly decreases; The temporary plugging pressure increase rate of particles increased from 27.27% to 34.49% and then slowly decreased. As the injection pressure increases, the pressure increase value and pressure increase rate in the coal fracture network temporary plugging by particles show a trend of first increasing and then decreasing. As the injection pressure increases, the decrease in fluid loss rate continues to increase from 0.034 cm^3^/s to 0.086 cm^3^/s; The reduction rate of fluid loss rate gradually decreased from 43.03% to 28.01%.

(3) Construct equations to describe the relationship between various parameters and fluid loss rate. Injection pressure has a significant impact on fluid loss rate, while fracture width and temporary plugging position have relatively small effects on fluid loss rate during temporary plugging fracturing. The fitting formula R^2^ is 0.988.

(4) Pressure difference is the driving force behind fluid flow. The flow velocity of the fluid in the coal fracture network depends on the pressure difference of the coal fracture network, and the different positions of the pores result in differences in the flow field of the fluid inside the pores.

(5) The particle temporary plugging pressure increase values, pressure increase rates, reduction values of the outlet volumetric flow rate, and reduction rates of the outlet volumetric flow rate obtained from the experimental results are consistent with the numerical model results.

(6) This study can provide scientific basis and data support for the application of temporary plugging and fracturing technology in coal to increase the extraction of coal seam gas, and to address the issues of particle transport and temporary plugging pressure control.

**Question8.** The manuscript contains numerous grammatical errors, awkward expressions, and unclear sentences. The authors should carefully revise the entire manuscript to improve readability, clarity, and academic expression. Professional English editing is recommended to ensure that the technical content is communicated accurately and effectively.

**Answer:** The expressions in the paper have been revised in accordance with expert opinions, as demonstrated in **Fig. 4**.

**Fig.4.** Proof about proofreading
