# Supplementary material for: Numerical simulation study on pressure response and fluid loss mechanism of coal fracture network under particle temporary plugging
Source: PLoS One. 2026 Jul 24;21(7):e0352681. doi: 10.1371/journal.pone.0352681 (PMC13399314; doi:10.1371/journal.pone.0352681)
Supplement: Peer review history

# Peer review: Decision Letter 0

**DOI:** 10.1371/journal.pone.0352681.r001

**Authors:** Xinyuan Gao

28 Jun 2026

PONE-D-26-28982

Numerical simulation study on pressure response and fluid loss mechanism of coal fracture network under particle temporary plugging

PLOS One

Dear Dr. Chen,

Thank you for submitting your manuscript to PLOS ONE. After careful consideration, we feel that it has merit but does not fully meet PLOS ONE’s publication criteria as it currently stands. Therefore, we invite you to submit a revised version of the manuscript that addresses the points raised during the review process.

Please submit your revised manuscript by Aug 27 2026 11:59PM. If you will need more time than this to complete your revisions, please reply to this message or contact the journal office at plosone@plos.org. When you're ready to submit your revision, log on to https://www.editorialmanager.com/pone/ and select the 'Submissions Needing Revision' folder to locate your manuscript file.

Please include the following items when submitting your revised manuscript:

- A letter that responds to each point raised by the academic editor and reviewer(s). You should upload this letter as a separate file labeled 'Response to Reviewers'.
- A marked-up copy of your manuscript that highlights changes made to the original version. You should upload this as a separate file labeled 'Revised Manuscript with Track Changes'.
- An unmarked version of your revised paper without tracked changes. You should upload this as a separate file labeled 'Manuscript'.

If you would like to make changes to your financial disclosure, please include your updated statement in your cover letter. Guidelines for resubmitting your figure files are available below the reviewer comments at the end of this letter.

If applicable, we recommend that you deposit your laboratory protocols in protocols.io to enhance the reproducibility of your results. Protocols.io assigns your protocol its own identifier (DOI) so that it can be cited independently in the future. For instructions see: https://journals.plos.org/plosone/s/submission-guidelines#loc-laboratory-protocols. Additionally, PLOS ONE offers an option for publishing peer-reviewed Lab Protocol articles, which describe protocols hosted on protocols.io. Read more information on sharing protocols at https://plos.org/protocols?utm_medium=editorial-email&utm_source=authorletters&utm_campaign=protocols.

As the corresponding author, your ORCID iD is verified in the submission system and will appear in the published article. PLOS supports the use of ORCID, and we encourage all coauthors to register for an ORCID iD and use it as well. Please encourage your coauthors to verify their ORCID iD within the submission system before final acceptance, as unverified ORCID iDs will not appear in the published article. *Only*  the individual author can complete the verification step; PLOS staff *cannot*  verify ORCID iDs on behalf of authors.

We look forward to receiving your revised manuscript.

Kind regards,

Xinyuan Gao

Academic Editor

PLOS One

Journal Requirements:

When submitting your revision, we need you to address these additional requirements.

1.Please ensure that your manuscript meets PLOS ONE's style requirements, including those for file naming. The PLOS ONE style templates can be found at

https://journals.plos.org/plosone/s/file?id=wjVg/PLOSOne_formatting_sample_main_body.pdf and

https://journals.plos.org/plosone/s/file?id=ba62/PLOSOne_formatting_sample_title_authors_affiliations.pdf

2. Please note that funding information should not appear in any section or other areas of your manuscript. We will only publish funding information present in the Funding Statement section of the online submission form. Please remove any funding-related text from the manuscript.

3. We note that the grant information you provided in the ‘Funding Information’ and ‘Financial Disclosure’ sections do not match.

When you resubmit, please ensure that you provide the correct grant numbers for the awards you received for your study in the ‘Funding Information’ section.

4. When completing the data availability statement of the submission form, you indicated that you will make your data available on acceptance. We strongly recommend all authors decide on a data sharing plan before acceptance, as the process can be lengthy and hold up publication timelines. Please note that, though access restrictions are acceptable now, your entire data will need to be made freely accessible if your manuscript is accepted for publication. This policy applies to all data except where public deposition would breach compliance with the protocol approved by your research ethics board. If you are unable to adhere to our open data policy, please kindly revise your statement to explain your reasoning and we will seek the editor's input on an exemption. Please be assured that, once you have provided your new statement, the assessment of your exemption will not hold up the peer review process.

5. PLOS requires an ORCID iD for the corresponding author in Editorial Manager on papers submitted after December 6th, 2016. Please ensure that you have an ORCID iD and that it is validated in Editorial Manager. To do this, go to ‘Update my Information’ (in the upper left-hand corner of the main menu), and click on the Fetch/Validate link next to the ORCID field. This will take you to the ORCID site and allow you to create a new iD or authenticate a pre-existing iD in Editorial Manager.

6. We note that Figure 3 in your submission contain copyrighted images. All PLOS content is published under the Creative Commons Attribution License (CC BY 4.0), which means that the manuscript, images, and Supporting Information files will be freely available online, and any third party is permitted to access, download, copy, distribute, and use these materials in any way, even commercially, with proper attribution. For more information, see our copyright guidelines: http://journals.plos.org/plosone/s/licenses-and-copyright.

We require you to either (1) present written permission from the copyright holder to publish these figures specifically under the CC BY 4.0 license, or (2) remove the figures from your submission:

1. You may seek permission from the original copyright holder of Figure 3 to publish the content specifically under the CC BY 4.0 license.

We recommend that you contact the original copyright holder with the Content Permission Form (http://journals.plos.org/plosone/s/file?id=7c09/content-permission-form.pdf) and the following text:

“I request permission for the open-access journal PLOS ONE to publish XXX under the Creative Commons Attribution License (CCAL) CC BY 4.0 (http://creativecommons.org/licenses/by/4.0/). Please be aware that this license allows unrestricted use and distribution, even commercially, by third parties. Please reply and provide explicit written permission to publish XXX under a CC BY license and complete the attached form.”

Please upload the completed Content Permission Form or other proof of granted permissions as an "Other" file with your submission.

In the figure caption of the copyrighted figure, please include the following text: “Reprinted from [ref] under a CC BY license, with permission from [name of publisher], original copyright [original copyright year].”

2. If you are unable to obtain permission from the original copyright holder to publish these figures under the CC BY 4.0 license or if the copyright holder’s requirements are incompatible with the CC BY 4.0 license, please either i) remove the figure or ii) supply a replacement figure that complies with the CC BY 4.0 license. Please check copyright information on all replacement figures and update the figure caption with source information. If applicable, please specify in the figure caption text when a figure is similar but not identical to the original image and is therefore for illustrative purposes only.

7. If the reviewer comments include a recommendation to cite specific previously published works, please review and evaluate these publications to determine whether they are relevant and should be cited. There is no requirement to cite these works unless the editor has indicated otherwise.

[Note: HTML markup is below. Please do not edit.]

Reviewers' comments:

Reviewer's Responses to Questions

**Comments to the Author**

1. Is the manuscript technically sound, and do the data support the conclusions?

The manuscript must describe a technically sound piece of scientific research with data that supports the conclusions. Experiments must have been conducted rigorously, with appropriate controls, replication, and sample sizes. The conclusions must be drawn appropriately based on the data presented.

Reviewer #1: Yes

Reviewer #2: Yes

**********

2. Has the statistical analysis been performed appropriately and rigorously?

Reviewer #1: Yes

Reviewer #2: Yes

**********

3. Have the authors made all data underlying the findings in their manuscript fully available?

The PLOS Data policy requires authors to make all data underlying the findings described in their manuscript fully available without restriction, with rare exception (please refer to the Data Availability Statement in the manuscript PDF file). The data should be provided as part of the manuscript or its supporting information, or deposited to a public repository. For example, in addition to summary statistics, the data points behind means, medians and variance measures should be available. If there are restrictions on publicly sharing data—e.g. participant privacy or use of data from a third party—those must be specified.

Reviewer #1: Yes

Reviewer #2: Yes

**********

4. Is the manuscript presented in an intelligible fashion and written in standard English?

PLOS ONE does not copyedit accepted manuscripts, so the language in submitted articles must be clear, correct, and unambiguous. Any typographical or grammatical errors should be corrected at revision, so please note any specific errors here.

Reviewer #1: Yes

Reviewer #2: No

**********

5. Review Comments to the Author

Please use the space provided to explain your answers to the questions above. You may also include additional comments for the author, including concerns about dual publication, research ethics, or publication ethics. (Please upload your review as an attachment if it exceeds 20,000 characters)

Reviewer #1: 1. This manuscript does not mention a sensitivity analysis of the Formula�7� with respect to different input parameters.

2. The conclusion does not fully capture the significance of the model or the experimental findings. Consider revising it to better reflect the novelty and contributions of the work, as well as including key quantitative results.

3. Several similar but inconsistent expressions are used throughout the manuscript, including "temporary plugging ," "temporarily blocking," and "temporary blockage." In some cases, multiple expressions are used interchangeably even within the same context. The authors are advised to standardize the key terminology throughout the paper, especially in the title, abstract, methods, results, figure captions, and conclusions.

4. The English expression and academic writing specifications of the whole text still need to be systematically polished. There are some language and formatting problems in the current manuscript, such as long titles or improper collocations in some sentences. It is recommended that authors polish the title, abstract, introduction, conclusion and diagram description in English during the revision process, and unify the terminology, symbols and expression style.

5. The manuscript does not explain how the segmentation threshold was determined. Since these steps directly affect the realism of the geometric model, the authors should provide a more detailed description of the image-processing workflow.

Reviewer #2: This manuscript investigates temporary plugging fracturing using supercritical CO2 and flexible solidified particles generated by hot alkali water, with the aim of improving coalbed methane recovery and supporting integrated technologies for underground CO2 storage and gas emission control. The study combines CT-based reconstruction, microscopic characterisation of solidified particles, coupled fluid–solid numerical simulation, and laboratory experiments to examine pressure enhancement and fluid loss behaviour after temporary plugging in coal pores and fractures. The topic is important and worthy of investigation, as it addresses a relevant technical challenge in coalbed methane production, CO2 utilisation/storage, and hydraulic control of gas emissions. The manuscript has practical significance and presents useful findings; however, several minor revisions are required to improve clarity, presentation, and readability before publication.

1. The research gap in the abstract is not sufficiently clear and should be further refined. The authors are encouraged to revise the abstract following a more logical structure, including background, research gap, methodology, key results, and significance. In addition, the key findings should be highlighted more clearly and, where possible, supported by quantitative results. The current abstract contains useful information, but it is relatively long and would benefit from a more concise and focused presentation.

2. Some keywords may not be suitable for indexing the paper. For example, “connectivity of pores and fractures” and “temporary plugging with pressure increase” appear more like descriptive phrases than effective keywords. The authors should revise the keywords to better reflect the main topic, methods, materials, and application of the study. Potential keywords may include terms related to temporary plugging fracturing, supercritical CO₂, coalbed methane, fluid loss, pore–fracture structure, and coupled fluid–solid simulation.

3. The significance of the study should be further highlighted at the end of the Introduction. The authors should clearly explain how this work advances the understanding of temporary plugging mechanisms, pressure enhancement, and fluid loss behaviour in coal pores and fractures. The practical relevance to coalbed methane recovery, CO₂ storage, and gas emission control should also be emphasised.

4. The numerical modelling methodology requires further clarification. The authors should clearly state which simulation software was used, how the numerical model was constructed from the CT-based pore–fracture structure, and what assumptions were adopted in the coupled fluid–solid simulation. In addition, the determination of key simulation parameters should be explained in more detail, including their sources, units, and physical meanings.

5. It is suggested that the authors separate the experimental equipment from the experimental procedure to improve the clarity of the methodology section. In particular, the self-developed SC-CO₂–hot alkali injection test system should be introduced in greater detail, including its main components, operating conditions, measurement methods, and how it was used to validate the numerical simulation results.

6. There are some differences between the numerical simulation results and the laboratory experimental results. The authors should provide a clearer explanation and justification for these differences. Possible reasons may include simplifications in the numerical model, uncertainty in pore–fracture reconstruction, differences between idealised simulation conditions and experimental conditions, measurement errors, or limitations in parameter determination.

7. The conclusion should be revised to better highlight the key findings of the study. At present, the main contributions are not sufficiently emphasised. The authors should summarise the major findings regarding plugging location, fracture size, injection pressure, pressure enhancement, and fluid loss rate. The conclusion should also clearly state the scientific and practical implications of the work.

8. The manuscript contains numerous grammatical errors, awkward expressions, and unclear sentences. The authors should carefully revise the entire manuscript to improve readability, clarity, and academic expression. Professional English editing is recommended to ensure that the technical content is communicated accurately and effectively.

**********

6. PLOS authors have the option to publish the peer review history of their article (what does this mean?). If published, this will include your full peer review and any attached files.

If you choose “no”, your identity will remain anonymous but your review may still be made public.

**Do you want your identity to be public for this peer review?**  For information about this choice, including consent withdrawal, please see our Privacy Policy.

Reviewer #1: No

Reviewer #2: No

**********

[NOTE: If reviewer comments were submitted as an attachment file, they will be attached to this email and accessible via the submission site. Please log into your account, locate the manuscript record, and check for the action link "View Attachments". If this link does not appear, there are no attachment files.]

To ensure your figures meet our technical requirements, please review our figure guidelines: https://journals.plos.org/plosone/s/figures

You may also use PLOS’s free figure tool, NAAS, to help you prepare publication quality figures: https://journals.plos.org/plosone/s/figures#loc-tools-for-figure-preparation.

NAAS will assess whether your figures meet our technical requirements by comparing each figure against our figure specifications.

# Author comment: Author response to Decision Letter 1

**DOI:** 10.1371/journal.pone.0352681.r002

3 Jul 2026

Dear editors and reviewers:

We would like to express our gratitude to the esteemed experts for your valuable feedback. We have diligently revised the manuscript in accordance with the suggestions provided, and we hope that the paper will be accepted. Additionally, we extend our sincere appreciation to the editors and reviewers for your contributions to the advancement of scientific research !

Yours sincerely

Doctor Chen Jian

July 1, 2026

Explanations about Modifications

Reviewer 1#

Question1. This manuscript does not mention a sensitivity analysis of the model with respect to different input parameters.

Answer: We appreciate the expert feedback. An analysis of the influences of relevant parameters on the model has been added.

Where represents the fluid loss rate of the coal body after the temporary temporary plugging particles achieve stable temporary plugging, cm³/s; represents the average internal pressure of the coal body after the temporary temporary plugging particles achieve stable temporary plugging, MPa; represents the injection pressure, MPa; represents the distance from the temporary plugging position to the inlet, μm; represents the equivalent width of the fractures at the temporary plugging position, μm.

The modified content (Line 1-8, Page 34) is as follows:

Related parameters were selected for the sensitivity analysis, as illustrated in Fig. 1 (Fig. 10 in the paper, Line 1, Page 34). Steps 1 to 10 represent the ten selected data groups, each corresponding to different step lengths, reflecting the changes of each parameter at various values. The fluid loss rate decreases with the equivalent width of the fractures at the temporary plugging position, while it increases with increases in the injection pressure and distance from the temporary plugging position to the inlet. Moreover, the differences in the relative rate of change under different step lengths in Fig. 1 indicate that the influence of crack width and temporary plugging position on fluid loss during temporary plugging fracturing is relatively small. The injection pressure has a significant impact on the fluid loss rate. Meanwhile, under the same conditions of crack width, temporary plugging position, and injection pressure, there is a quadratic function relationship between the temporary plugging pressure and the fluid loss rate. The fitting formula R2 is 0.998. The numerical simulation calculation shows a good fit between the actual values and the predicted values of the fitting formula.

Fig. 1. Sensitivity analysis of relevant parameters

Question2. The conclusion does not fully capture the significance of the model or the experimental findings. Consider revising it to better reflect the novelty and contributions of the work, as well as including key quantitative results.

Answer: Based on expert feedback, the conclusion has been revised to include descriptions of quantitative results (Lines 5-21, Page 45; Lines 1-18, Page 46).

The modified conclusion is as follows:

Conclusion:

(1) The distance between the temporary plugging position of particles and the injection port, as well as the number of blocked fractures, affect the pressure distribution, average pressure, fluid flow distribution, and fluid loss rate of the coal fracture network. When particles are blocked at different positions, there are differences in the average pressure of the fracture network, with a maximum of 12.03 MPa and a minimum of 8.05 MPa. For temporary plugging positions close to the entrance, when the particles are blocked in a single position, the average pressure after blocking is lower than when the particles are blocked in multiple positions. The closer the temporary plugging position of the particles is to the injection port, the lower the average pressure, and the farther the temporary plugging position is from the injection port, the better the average boosting effect of temporary plugging fracturing. When particles are blocked at different positions, there are differences in fluid loss rate, with a maximum of 0.152 cm3/s and a minimum of 0.037 cm3/s. The closer the particle temporary plugging position is to the inlet, the lower the fluid loss rate after blockage. For temporary plugging position close to the inlet, when the particle is temporary plugging at a single position, the fluid loss rate after blockage is greater than when the particle is blocked at multiple positions.

(2) The injection pressure affects the pressure boosting and fluid loss effect. As the injection pressure increases, the temporary plugging pressure increase value of particles increases from 0.75 MPa to 3.23 MPa and then slowly decreases; The temporary plugging pressure increase rate of particles increased from 27.27% to 34.49% and then slowly decreased. As the injection pressure increases, the pressure increase value and pressure increase rate in the coal fracture network temporary plugging by particles show a trend of first increasing and then decreasing. As the injection pressure increases, the decrease in fluid loss rate continues to increase from 0.034 cm3/s to 0.086 cm3/s; The reduction rate of fluid loss rate gradually decreased from 43.03% to 28.01%.

(3) Construct equations to describe the relationship between various parameters and fluid loss rate. Injection pressure has a significant impact on fluid loss rate, while fracture width and temporary plugging position have relatively small effects on fluid loss rate during temporary plugging fracturing. The fitting formula R2 is 0.988.

(4) Pressure difference is the driving force behind fluid flow. The flow velocity of the fluid in the coal fracture network depends on the pressure difference of the coal fracture network, and the different positions of the pores result in differences in the flow field of the fluid inside the pores.

(5) The particle temporary plugging pressure increase values, pressure increase rates, reduction values of the outlet volumetric flow rate, and reduction rates of the outlet volumetric flow rate obtained from the experimental results are consistent with the numerical model results.

(6) This study can provide scientific basis and data support for the application of temporary plugging and fracturing technology in coal to increase the extraction of coal seam gas, and to address the issues of particle transport and temporary plugging pressure control.

Question3. Several similar but inconsistent expressions are used throughout the manuscript, including "temporary plugging ," "temporarily blocking," and "temporary blockage." In some cases, multiple expressions are used interchangeably even within the same context. The authors are advised to standardize the key terminology throughout the paper, especially in the title, abstract, methods, results, figure captions, and conclusions.

Answer: Thank you for the expert's revision suggestions. We have unified the key terms in the text. For example, "temporary plugging", "temporarily plugging", and "temporary blockage" have been unified as "temporary plugging".

Question4. The English expression and academic writing specifications of the whole text still need to be systematically polished. There are some language and formatting problems in the current manuscript, such as long titles or improper collocations in some sentences. It is recommended that authors polish the title, abstract, introduction, conclusion and diagram description in English during the revision process, and unify the terminology, symbols and expression style.

Answer: We appreciate the expert feedback.The expressions in the paper have been revised in accordance with expert opinions, as demonstrated in Fig. 2

Fig.2. Proof about proofreading

Question5. The manuscript does not explain how the segmentation threshold was determined. Since these steps directly affect the realism of the geometric model, the authors should provide a more detailed description of the image-processing workflow, parameter selection criteria, and representativeness verification.

Answer: Based on the expert's revision suggestions, additional explanations are provided for the CT scan image processing process. The modified content (Line 7-18, Page 7; Line 1-2, Page 8) is as follows:

The CT scanning experiment obtained a two-dimensional CT grayscale image of the coal sample, which was imported into Avizo for processing. Image processing techniques such as filtering, noise reduction, and threshold segmentation were applied to obtain a binary image.

The ultimate goal of noise reduction is to provide high signal-to-noise ratio data for coal and rock fracture network modeling, while avoiding the loss of microstructural information due to excessive smoothing. Selecting a median filter to achieve image denoising not only removes noise from the image but also preserves the edge features of the image, resulting in good denoising and image restoration effects. Based on the bimodal distribution characteristics of the two-phase grayscale histogram of coal rock matrix and pores, a globally optimal bimodal adaptive threshold iteration algorithm is employed, combined with cross-checking of multiple slices within the same batch, to determine the segmentation threshold. Avoid the upper and lower end faces of the coal sample, the edge processing disturbance zone, and the local abnormal high-density mineral inclusion zone. Select typical cross sections from the upper, middle, and lower layers at equal intervals along the axial direction of the coal sample. Images with obvious fracture structures were selected for processing, and the final geometric structure of the micro-macroscopic connected pores and fractures of coal was obtained, as shown in Fig. 3 (Fig. 1 in the paper, Line 5, Page 8).

Fig. 3. Geometric Model Construction Process

Reviewer 2#

Question1. The research gap in the abstract is not sufficiently clear and should be further refined. The authors are encouraged to revise the abstract following a more logical structure, including background, research gap, methodology, key results, and significance. In addition, the key findings should be highlighted more clearly and, where possible, supported by quantitative results. The current abstract contains useful information, but it is relatively long and would benefit from a more concise and focused presentation.

Answer: Thank you for the expert's revision suggestions. Based on expert feedback, the abstract has been revised to include descriptions of quantitative results (Lines 1-22, Page 2).

The modified abstract is as follows:

Abstract: To address the issue of fluid loss leading to pressure failure when applying temporary plugging fracturing technology from the oil industry to enhance gas recovery in coal mines, and considering the current low efficiency of CO2 solidification and utilization, a new method is proposed to inject SC-CO2 and hot alkali solution together into coal to produce solidified particles as temporary plugging agents for solidification and utilization of CO2 and fracturing transformation of coal. At present, the mechanisms governing pressure increase during temporary plugging with solidified CO2 particles, as well as the relationship between internal pore and fracture pressures after plugging and fluid loss rate, remain unclear. Based on CT images, a complex geometric model of interconnected pores and fractures of coal is constructed. The morphology of solidified CO2 particles is obtained via microscopic scanning, and conducts numerical simulation research on fluid solid coupling. Clearly define the pressure and flow distribution inside the coal under different temporary plugging positions and injection conditions. Revealing the pressure and fluid loss law inside the coal after temporary plugging. The results show that when particles are temporary plugging at different positions, the average internal pressure in the coal ranges from 8.05 MPa to 12.03 MPa. The fluid loss rate varies from 0.037 cm3/s to 0.152 cm3/s. Establish equations for the relationship between injection pressure, fracture size, temporary plugging location, internal pressure, and fluid loss rate, with a fitting formula R2 of 0.988. Furthermore, an experimental setup and methodology involving supercritical CO2 and hot alkali liquor injection were designed to validate the accuracy of the numerical simulation results regarding pressure enhancement and fluid loss under solidified particle plugging. This study provides theoretical support for enhanced coalbed methane recovery and the development of integrated technologies for underground CO2 storage and hydraulic control of gas emissions.

Question2. Some keywords may not be suitable for indexing the paper. For example, “connectivity of pores and fractures” and “temporary plugging with pressure increase” appear more like descriptive phrases than effective keywords. The authors should revise the keywords to better reflect the main topic, methods, materials, and application of the study. Potential keywords may include terms related to temporary plugging fracturing, supercritical CO2, coalbed methane, fluid loss, pore–fracture structure, and coupled fluid–solid simulation.

Answer: Based on expert feedback, the keywords (Lines 1-2, Page 3) has been modified as follows:

Keywords: Temporary plugging fracturing; Interconnected pore and fracture; Pressure response; Fluid loss rate.

Question3. The significance of the study should be further highlighted at the end of the Introduction. The authors should clearly explain how this work advances the understanding of temporary plugging mechanisms, pressure enhancement, and fluid loss behaviour in coal pores and fractures. The practical relevance to coalbed methane recovery, CO2 storage, and gas emission control should also be emphasised.

Answer: Thank you for the expert's revision suggestions. The introduction has been modified as follows:

(Lines 4-7, Page 3)

Coalbed methane is a coal related energy source with abundant resources. Coal reservoirs generally have characteristics such as low permeability, high adsorption, and strong heterogeneity. Traditional hydraulic fracturing technology faces many challenges in coal reservoir transformation and gas extraction.

(Lines 19-21, Page 3; Lines 1-5, Page 4)

The resource utilization and storage of CO2 has become an important path to achieve the “dual carbon” strategic goals. Supercritical CO2 (SC-CO2) has low viscosity, strong permeability, and good injection effect in coal seams, and shows unique advantages in fields such as coal seam gas displacement and coal reservoir fracturing modification. However, the existing SC-CO2 fracturing technology mainly focuses on its physical and mechanical effects as a fracturing medium, and pays insufficient attention to the solidification and utilization of CO2 after injection into coal rock. The efficiency of CO2 solidification and storage is low, and the potential of CO2 resource utilization in the fracturing process has not been fully exploited.

(Lines 13-15, Page 6)

This study can provide scientific basis and data support for the temporary plugging and pressure control issues faced by coal mines in applying temporary plugging and fracturing technology to increase coalbed methane production.

Question4. The numerical modelling methodology requires further clarification. The authors should clearly state which simulation software was used, how the numerical model was constructed from the CT-based pore–fracture structure, and what assumptions were adopted in the coupled fluid–solid simulation. In addition, the determination of key simulation parameters should be explained in more detail, including their sources, units, and physical meanings.

Answer: Thank you for the expert's revision suggestions. The numerical modelling methodology has been clarified as follows:

(Line 7-18, Page 7; Line 1-2, Page 8)

The CT scanning experiment obtained a two-dimensional CT grayscale image of the coal sample, which was imported into Avizo for processing. Image processing techniques such as filtering, noise reduction, and threshold segmentation were applied to obtain a binary image.

The ultimate goal of noise reduction is to provide high signal-to-noise ratio data for coal and rock fracture network modeling, while avoiding t

Attachment Submitted filename: Response to Reviewers.doc

# Peer review: Decision Letter 1

**DOI:** 10.1371/journal.pone.0352681.r003

**Authors:** Xinyuan Gao

9 Jul 2026

Numerical simulation study on pressure response and fluid loss mechanism of coal fracture network under particle temporary plugging

PONE-D-26-28982R1

Dear Dr. Chen,

We’re pleased to inform you that your manuscript has been judged scientifically suitable for publication and will be formally accepted for publication once it meets all outstanding technical requirements.

Within one week, you’ll receive an e-mail detailing the required amendments. When these have been addressed, you’ll receive a formal acceptance letter and your manuscript will be scheduled for publication.

An invoice will be generated when your article is formally accepted. Please note, if your institution has a publishing partnership with PLOS and your article meets the relevant criteria, all or part of your publication costs will be covered. Please make sure your user information is up-to-date by logging into Editorial Manager at Editorial Manager® and clicking the ‘Update My Information' link at the top of the page. For questions related to billing, please contact billing support.

If your institution or institutions have a press office, please notify them about your upcoming paper to help maximize its impact. If they’ll be preparing press materials, please inform our press team as soon as possible -- no later than 48 hours after receiving the formal acceptance. Your manuscript will remain under strict press embargo until 2 pm Eastern Time on the date of publication. For more information, please contact onepress@plos.org.

Kind regards,

Xinyuan Gao

Academic Editor

PLOS One

Additional Editor Comments (optional):

Reviewers' comments:

Reviewer's Responses to Questions

**Comments to the Author**

1. If the authors have adequately addressed your comments raised in a previous round of review and you feel that this manuscript is now acceptable for publication, you may indicate that here to bypass the “Comments to the Author” section, enter your conflict of interest statement in the “Confidential to Editor” section, and submit your "Accept" recommendation.

Reviewer #1: All comments have been addressed

Reviewer #2: All comments have been addressed

**********

2. Is the manuscript technically sound, and do the data support the conclusions?

The manuscript must describe a technically sound piece of scientific research with data that supports the conclusions. Experiments must have been conducted rigorously, with appropriate controls, replication, and sample sizes. The conclusions must be drawn appropriately based on the data presented.

Reviewer #1: Yes

Reviewer #2: Yes

**********

3. Has the statistical analysis been performed appropriately and rigorously?

Reviewer #1: Yes

Reviewer #2: Yes

**********

4. Have the authors made all data underlying the findings in their manuscript fully available?

The PLOS Data policy requires authors to make all data underlying the findings described in their manuscript fully available without restriction, with rare exception (please refer to the Data Availability Statement in the manuscript PDF file). The data should be provided as part of the manuscript or its supporting information, or deposited to a public repository. For example, in addition to summary statistics, the data points behind means, medians and variance measures should be available. If there are restrictions on publicly sharing data—e.g. participant privacy or use of data from a third party—those must be specified.

Reviewer #1: Yes

Reviewer #2: Yes

**********

5. Is the manuscript presented in an intelligible fashion and written in standard English?

PLOS ONE does not copyedit accepted manuscripts, so the language in submitted articles must be clear, correct, and unambiguous. Any typographical or grammatical errors should be corrected at revision, so please note any specific errors here.

Reviewer #1: Yes

Reviewer #2: Yes

**********

6. Review Comments to the Author

Please use the space provided to explain your answers to the questions above. You may also include additional comments for the author, including concerns about dual publication, research ethics, or publication ethics. (Please upload your review as an attachment if it exceeds 20,000 characters)

Reviewer #1: The authors have answered my questions well and revisions have been made carefully. This paper can be published as it is.

Reviewer #2: I am satisfied with all revisions made by authors and the paper meets the requirement of the Journal PLOS One.

**********

7. PLOS authors have the option to publish the peer review history of their article (what does this mean?). If published, this will include your full peer review and any attached files.

If you choose “no”, your identity will remain anonymous but your review may still be made public.

**Do you want your identity to be public for this peer review?**  For information about this choice, including consent withdrawal, please see our Privacy Policy.

Reviewer #1: No

Reviewer #2: No

**********

# Editor report: Acceptance letter

**DOI:** 10.1371/journal.pone.0352681.r004

**Authors:** Xinyuan Gao

PONE-D-26-28982R1

PLOS One

Dear Dr. Chen,

I'm pleased to inform you that your manuscript has been deemed suitable for publication in PLOS One. Congratulations! Your manuscript is now being handed over to our production team.

At this stage, our production department will prepare your paper for publication. This includes ensuring the following:

* All references, tables, and figures are properly cited

* All relevant supporting information is included in the manuscript submission,

* There are no issues that prevent the paper from being properly typeset

You will receive further instructions from the production team, including instructions on how to review your proof when it is ready. Please keep in mind that we are working through a large volume of accepted articles, so please give us a few days to review your paper and let you know the next and final steps.

Lastly, if your institution or institutions have a press office, please let them know about your upcoming paper now to help maximize its impact. If they'll be preparing press materials, please inform our press team within the next 48 hours. Your manuscript will remain under strict press embargo until 2 pm Eastern Time on the date of publication. For more information, please contact onepress@plos.org.

You will receive an invoice from PLOS for your publication fee after your manuscript has reached the completed accept phase. If you receive an email requesting payment before acceptance or for any other service, this may be a phishing scheme. Learn how to identify phishing emails and protect your accounts at https://explore.plos.org/phishing.

If we can help with anything else, please email us at customercare@plos.org.

Thank you for submitting your work to PLOS ONE and supporting open access.

Kind regards,

PLOS ONE Editorial Office Staff

on behalf of

Dr. Xinyuan Gao

Academic Editor

PLOS One
